# Homeostatic Synaptic Plasticity of Miniature Excitatory Postsynaptic Currents in Mouse Cortical Cultures Requires Neuronal Rab3A

**DOI:** 10.1101/2023.06.14.544980

**Published:** 2024-07-17

**Authors:** Andrew G. Koesters, Mark M. Rich, Kathrin L. Engisch

**Affiliations:** 1 Department of Pharmacology and Systems Physiology, University of Cincinnati College of Medicine, Cincinnati, OH 45267, USA; 2 Department of Neuroscience, Cell Biology and Physiology, Boonshoft School of Medicine, Wright State University, Dayton, OH 45345; 3 Department of Neuroscience, Cell Biology and Physiology, Boonshoft School of Medicine and the College of Science and Mathematics, Wright State University, Dayton, OH 45435

**Keywords:** Homeostatic Synaptic Plasticity, Synaptic Scaling, mEPSCs, Rab3A, AMPA receptors

## Abstract

Following prolonged activity blockade, amplitudes of miniature excitatory postsynaptic currents (mEPSCs) increase, a form of plasticity termed “homeostatic synaptic plasticity.” We previously showed that a presynaptic protein, the small GTPase Rab3A, is required for full expression of the increase in miniature endplate current amplitudes following prolonged blockade of action potential activity at the mouse neuromuscular junction in vivo (Wang et al., 2011), but it is unknown whether this form of Rab3A-dependent homeostatic plasticity shares any characteristics with central synapses. We show here that homeostatic synaptic plasticity of mEPSCs is impaired in mouse cortical neuron cultures prepared from Rab3A^−/−^ and mutant mice expressing a single point mutation of Rab3A, Rab3A Earlybird mice. To determine if Rab3A is involved in the well-established homeostatic increase in postsynaptic AMPA-type receptors (AMPARs), we performed a series of experiments in which electrophysiological recordings of mEPSCs and confocal imaging of synaptic AMPAR immunofluorescence were assessed within the same cultures. We found that Rab3A was required for the increase in synaptic AMPARs following prolonged activity blockade, but the increase in mEPSC amplitudes was not always accompanied by an increase in postsynaptic AMPAR levels, suggesting other factors may contribute. Finally, we demonstrate that Rab3A is acting in neurons because only selective loss of Rab3A in neurons, not glia, disrupted the homeostatic increase in mEPSC amplitudes. This is the first demonstration that neuronal Rab3A is required for homeostatic synaptic plasticity and that it does so partially through regulation of the surface expression of AMPA receptors.

## Introduction

One of the most studied phenomena triggered by prolonged activity blockade is the increase in amplitudes of miniature excitatory postsynaptic currents (mEPSCs) in neurons. First demonstrated in cultures of dissociated cortical neurons (Turrigiano et al., 1998) and spinal cord neurons (O’Brien et al., 1998), the compensatory response to prolonged loss of activity has been dubbed “homeostatic synaptic plasticity” (Turrigiano and Nelson, 2004; Pozo and Goda, 2010). These first two studies provided evidence that glutamate receptors were also increased after inactivity, via an increased response to exogenously applied glutamate (Turrigiano et al., 1998) and increased immunofluorescent labeling of GluA1 receptors at synaptic sites (O’Brien et al., 1998). The accompanying increase in synaptic AMPA receptors has been confirmed with immunofluorescence in multiple studies of hippocampal and cortical cultures treated with TTX (Wierenga et al., 2005; Hou et al., 2008; Ibata et al., 2008; Jakawich et al., 2010a; Tatavarty et al., 2013; Xu and Pozzo-Miller, 2017; Dubes et al., 2022). It is now well accepted that the homeostatic increase in mEPSC amplitudes in neurons is due to an increase in postsynaptic AMPA receptors.

In our previous work, we found that a TTX cuff applied for 48 hr around the sciatic nerve in mice led to an increase in the amplitude of the miniature endplate currents (mEPCs) recorded in tibialis anterior muscles. Surprisingly, and unlike central synapses, we could find no evidence that the acetylcholine receptors levels at the neuromuscular junction (NMJ) were increased in the TTX-cuff treated mice (Wang et al., 2005). This result led us to search for presynaptic molecules that might homeostatically regulate the presynaptic quantum. In previous studies in chromaffin cells, we identified the small GTPase Rab3A, a presynaptic vesicle protein, as a regulator of synaptic vesicle fusion pore opening (Wang et al., 2008), so we examined whether deletion of Rab3A (Rab3A^−/−^) might prevent homeostatic upregulation of mEPC amplitude at the NMJ. The results were clear: the homeostatic increase in mEPC amplitude induced by the TTX cuff was strongly reduced in the Rab3A^−/−^ mouse, and was completely abolished in the Earlybird mutant (Rab3A^*Ebd/Ebd*^), which has a single point mutation in Rab3A that causes a shift towards early awakening that is more dramatic than that of the Rab3A deletion mouse (Kapfhamer et al., 2002; Wang et al., 2011). These results led us to conclude that the homeostatic increase in mEPC amplitude at the NMJ is a presynaptic phenomenon.

It has remained somewhat of a mystery what the role of Rab3A is in synaptic transmission. The Rab3A^−/−^ mouse has minimal phenotypic abnormalities, with evoked synaptic transmission and mEPSCs essentially normal in hippocampal slices (Geppert et al., 1994). At the Rab3A^−/−^ NMJ, reductions in evoked transmission were detected, but only under conditions of reduced extracellular calcium (Coleman et al., 2007). There are some modest changes in short term plasticity during repetitive stimulus trains, with increased depression observed in response to moderate frequencies in hippocampal slices (Geppert et al., 1994) and at the mammalian NMJ (Coleman and Bykhovskaia, 2009), but increased facilitation in response to high frequency stimulation at the mammalian NMJ (Coleman and Bykhovskaia, 2010). The most dramatic effect of loss of Rab3A is the abolishment of a presynaptic form of long-term potentiation (LTP) at the mossy fiber-CA3 synapse that is induced by 25 Hz stimulation in the presence of NMDA blockers (Weisskopf et al., 1994; Castillo et al., 1997). Our result that prolonged inactivity-induced plasticity of mEPC amplitude at the mammalian NMJ is disrupted with loss of function of Rab3A further cements a role for Rab3A in activity-dependent plasticity of synaptic strength. However, a crucial question that remains is whether the TTX-cuff induced effect on the amplitude of the spontaneous synaptic current at the NMJ shares underlying mechanisms with central synapses. We set out to answer this question for the more well-studied phenomenon of homeostatic plasticity of mEPSCs in dissociated mouse cortical neurons prepared from wild type, Rab3A^−/−^ mice and Rab3a^*Ebd/Ebd*^ mice treated with TTX for 48 hr. We show here that homeostatic synaptic plasticity of mEPSC amplitude was strongly reduced in cultures from Rab3A^−/−^ mice, and completely abolished in cultures from Rab3A^*Ebd/Ebd*^ mice, supporting a shared mechanism with the neuromuscular junction. Unexpectedly, the modest increase in GluA2 immunofluorescence we observed in wild type cultures was not present in Rab3A^−/−^ cultures. Loss of Rab3A in neurons, but not in glia, disrupted the increase in mEPSC amplitudes after TTX treatment. These results support the idea that the increase in mEPSC amplitude is due to more postsynaptic receptors, and that neuronal Rab3A is necessary for this regulation. However, we found that GluA2 receptors and mEPSC amplitudes did not always change in parallel. This mismatch suggests there may be contributors other than postsynaptic receptor numbers to the homeostatic plasticity of mEPSC amplitude.

## Results

We previously reported that mixed cultures of cortical neurons and glia prepared from postnatal day 0–2 mouse pups responded to a block of action potential-mediated activity by a 48 hr TTX treatment with an increase in mEPSC amplitude (Hanes et al., 2020). Here, we asked the question whether cortical cultures prepared from mice lacking the small GTPase Rab3A, or, expressing a point mutation of Rab3A, Rab3A Earlybird, have an altered homeostatic plasticity response to a loss of network activity. The Rab3A Earlybird mutation was discovered in a mutagenesis screen in mice for genes involved in the rest-activity cycle (Kapfhamer et al., 2002). The Earlybird mutant was named for its phenotype, a significantly shorter circadian period which resulted in early awakening. In that study, the authors concluded that the Earlybird mutation was likely dominant-negative, as the shift in circadian period was substantially more dramatic than that observed in the Rab3A^−/−^ mice. As described in the Introduction, the effects of loss of Rab3A have been modest, so we included experiments using the Rab3A Earlybird mutant mice to see if there would be a more robust phenotype, and in doing so, strengthen conclusions based on the Rab3A deletion mice. To obtain Rab3A^−/−^ and Rab3A^*Ebd/Ebd*^ homozygotes, we established two mouse colonies of heterozygous breeders with cultures prepared from pups derived from a final breeding pair of homozygotes. Although we backcrossed Rab3A^+/−^ with Rab3A^+/*Ebd*^ for 11 generations, clear differences in mEPSC amplitudes in untreated cultures (see below) and in calcium current amplitudes in adrenal chromaffin cells (unpublished obs.) remained. Therefore, throughout this study we keep the two strains separate and there are two Rab3A^+/+^ or ‘wild type’ phenotypes.

**Figure 1.**
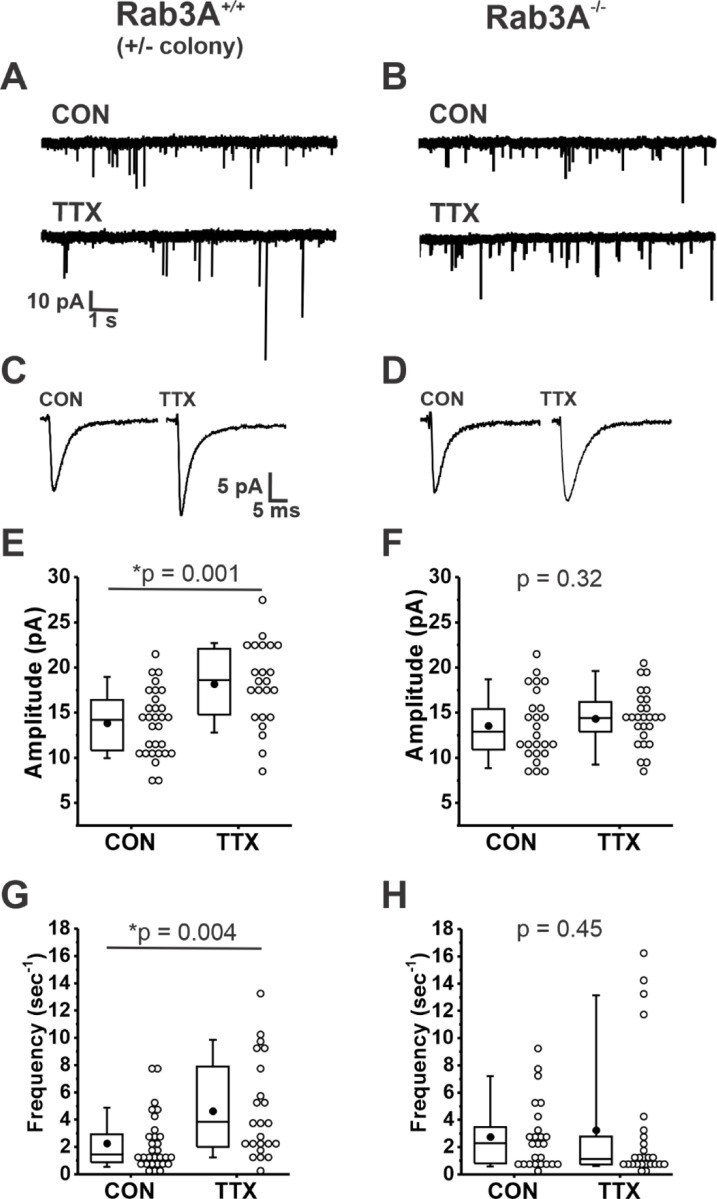
Loss of Rab3A prevented the TTX-induced increases in amplitudes and frequency of mEPSCs recorded in cultured mouse cortical neurons. (A) Ten second example traces recorded at −60 mV in pyramidal cortical neurons from an untreated (“CON”) and TTX-treated (“TTX”) neuron in cultures prepared from Rab3A^+/+^ mice from the Rab3A^+/−^ colony. (B) Ten second example traces recorded at −60 mV in pyramidal cortical neurons from an untreated (“CON”) and TTX-treated (“TTX”) neuron in cultures prepared from Rab3A^−/−^ mice. (C & D) Average traces for the recordings shown in A and B, respectively. (E) Box plots for average mEPSC amplitudes from untreated cells and TTX-treated cells in cultures prepared from Rab3A^+/+^ mice (Rab3A^+/−^ colony; CON, N = 30 cells, 13.9 ± 0.7 pA; TTX, N = 23 cells, 18.2 ± 0.9 pA; from 11 cultures). (F) Box plots for average mEPSC amplitudes from untreated cells and TTX-treated cells in cultures prepared from Rab3A^−/−^ mice (CON, N = 25 cells, 13.6 ± 0.7 pA; TTX, N = 26 cells, 14.3 ± 0.6 pA); from 11 cultures. (G) Box plots for average mEPSC frequency for same Rab3A^+/+^ cells as in (E) (CON, 2.26 ± 0.37 sec^−1^; TTX, 4.62 ± 0.74 sec^−1^). (H) Box plots for average mEPSC frequency for same Rab3A^−/−^cells as in (F) (CON, 2.74 ± 0.49 sec^−1^; TTX, 3.23 ± 0.93 sec^−1^). Box plot parameters: box ends, 25th and 75th percentiles; whiskers, 10th and 90th percentiles; open circles represent means from individual cells; line, median; dot, mean. p values (shown on the graphs) are from Kruskal-Wallis test. For all p-values, * with underline indicates significance with p < 0.05.

We found that loss of Rab3A resulted in a greatly impaired homeostatic increase after activity blockade. Example current traces of spontaneously occurring mEPSCs recorded from pyramidal neurons in untreated (CON) 13–14 DIV cortical cultures and sister cultures treated with 500 nM TTX for 48 hr prepared from wild type and Rab3A^−/−^ mice in the Rab3A^+/−^ colony are shown in Figures 1A and B, respectively. Average mEPSC waveforms from the same recordings are shown in Figures 1C and D. The mean mEPSC amplitudes for 30 control and 23 TTX-treated neurons from Rab3A^+/+^ cultures are displayed in the box and whisker plot in Figure 1E; after activity blockade the average mEPSC amplitude increased from 13.9 ± 0.7 pA to 18.2 ± 0.9 pA. In contrast, in cultures prepared from Rab3A^−/−^ mice, the average mEPSC amplitude was not significantly increased, for 25 untreated cells and 26 TTX-treated cells (Figure 1F, 13.6 ± 0.1 pA vs. 14.3 ± 0.6 pA). We found that TTX treatment also resulted in an increase in mEPSC frequency in cultures prepared from Rab3A^+/+^ mice, as shown in Figure 1G (CON, 2.26 ± 0.37 sec^−1^; TTX, 4.62 ± 0.74 sec^−1^). This TTX-induced frequency increase was strongly reduced in cultures from Rab3A^−/−^ mice, but there were a few outliers with very high frequencies still present after TTX treatment (Figure 1H, CON, 2.74 ± 0.49 Hz; TTX, 3.23 ± 0.93 Hz).

**Figure 2.**
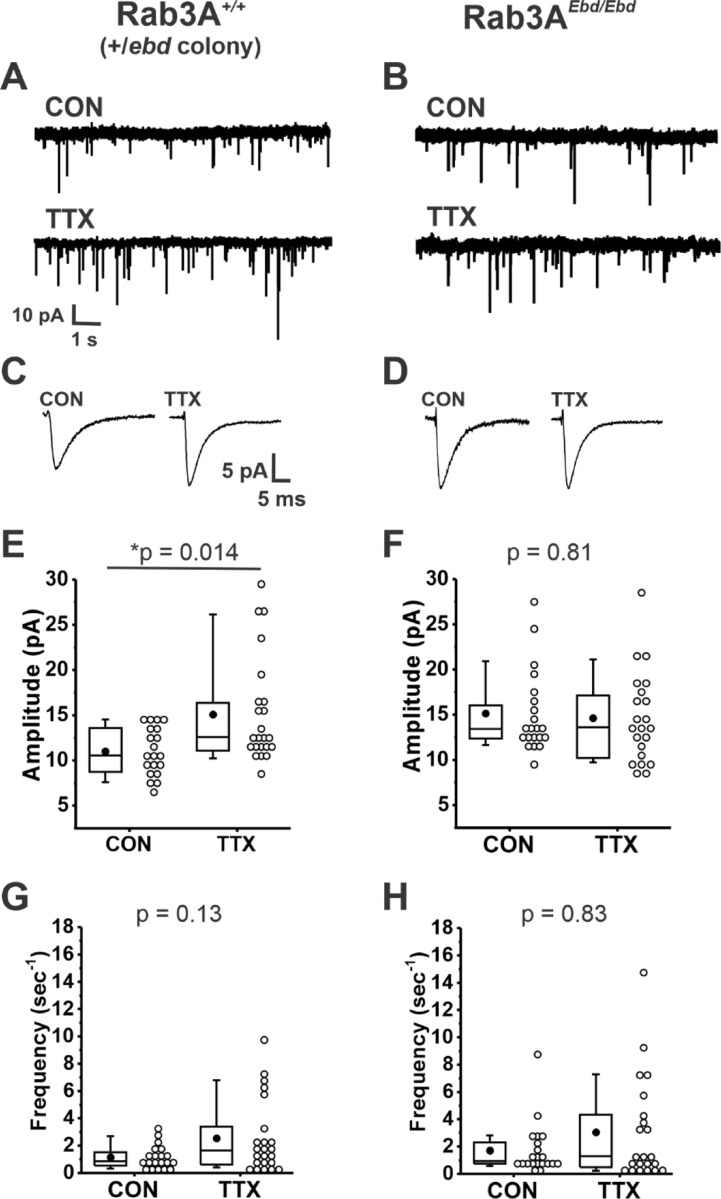
Normally functioning Rab3A was required for TTX-induced homeostatic plasticity of mEPSC amplitudes in cultured mouse cortical neurons. (A) Ten second example traces recorded at −60 mV in pyramidal cortical neurons from an untreated (“CON”) and TTX-treated (“TTX”) neuron in cultures prepared from Rab3A^+/+^ mice from the Rab3A^+/*Ebd*^ colony. (B) Ten second example traces recorded at −60 mV in pyramidal cortical neurons from an untreated (“CON”) and TTX-treated (“TTX”) neuron in cultures prepared from Rab3A^*Ebd/Ebd*^ mice. (C & D) Average traces for the recordings shown in A and B, respectively. (E) Box plots for average mEPSC amplitudes from untreated cells and TTX-treated cells from cultures prepared from Rab3A^+/+^ mice (Rab3A^+/*Ebd*^ colony; CON, N = 20 cells, 11.0 ± 0.6 pA; TTX, N = 23 cells, 15.1 ± 1.2 pA; from 6 cultures). (F) Box plots for average mEPSC amplitudes from untreated cells and TTX-treated cells from cultures prepared from Rab3A^*Ebd/Ebd*^ mice (CON, N = 21 cells, 15.1 ± 1.0 pA; TTX, N = 22 cells, 14.6 ± 1.1 pA; from 7 cultures.) (G) Box plots for average mEPSC frequency for same Rab3A^+/+^ cells as in (E) (CON, 1.15 ± 0.19 sec^−1^; TTX, 2.54 ± 0.55 sec^−1^). (H) Box plots for average mEPSC frequency for same Rab3A^*Ebd/Ebd*^ cells as in (F) (CON, 1.71 ± 0.41 sec^−1^; TTX, 3.05 ± 0.80 sec^−1^). Box plot parameters: box ends, 25th and 75th percentiles; whiskers, 10th and 90th percentiles; open circles represent means from individual cells; line, median; dot, mean. p values (shown on the graphs) are from Kruskal-Wallis test. For all p-values, * with underline indicates significance with p < 0.05.

We next examined homeostatic plasticity in cultures prepared from wild type mice in the Rab3A^+/*Ebd*^ colony. Example current traces of mEPSCs, and average mEPSC waveforms, are shown in Figures 2A and C, respectively, for cortical cultures prepared from wild type mice in the Rab3A^+/*Ebd*^ colony. Treatment with TTX for 48 hr led to a significant increase in the average mEPSC amplitude of 23 TTX-treated cells when compared to 20 untreated cells (Figure 2E, CON, ± 0.6 pA; TTX, 15.0 ± 1.3 pA). We note here that while the two wild type strains respond very similarly to activity blockade, the mean mEPSC amplitude in untreated cultures was significantly different in the two wild type strains, 13.9 ± 0.7 pA, wild type from Rab3A^+/−^ colony; ± 0.6 pA, wild type from Rab3A^+/*Ebd*^ colony (p = 0.004, Kruskal-Wallis test).

We found a complete disruption of homeostatic plasticity in cortical cultures prepared from Rab3A^*Ebd/Ebd*^ mice, as can been seen in viewing example mEPSC traces and average mEPSC waveforms (Figures 2B and D, respectively). The lack of TTX effect was confirmed in a comparison of mEPSC amplitude means for 21 untreated and 22 TTX-treated cells (Figure 2F, CON, 15.1 ± 1.0 pA vs. TTX, 14.6 ± 1.1 pA). There was a trend towards increased mEPSC frequency in TTX-treated cultures from this wild type strain, but the difference was not as robust as for the wild type cultures from Rab3A^+/−^ colony, and did not reach statistical significance (Figure 2G, CON, 1.15 ± 0.19 sec^−1^; TTX, 2.54 ± 0.55 sec^−1^). This trend was not observed in neurons from cultures prepared from Rab3A^*Ebd/Ebd*^ mice, likely due to an increase in frequency in untreated cells—frequencies remained high in TTX-treated cells (Figure 2H, CON, 1.71 ± 0.41 sec^−1^; TTX, 3.05 ± 0.80 sec^−1^).

Our results show that the homeostatic increase in mEPSC amplitude after activity blockade is disrupted in both Rab3A^−/−^ and the Rab3A^*Ebd/Ebd*^ cortical neurons, strongly supporting a crucial role of functioning Rab3A in this process. However, it is important to note that the disruption differs for Rab3A^−/−^ and Rab3A^*Ebd/Ebd*^. In the Rab3A^−/−^ data set, mEPSCs from untreated cultures were indistinguishable from mEPSCs from Rab3A^+/+^ untreated cultures, demonstrating loss of Rab3A has no impact on basal activity mEPSC amplitudes, but the increase in mEPSC amplitudes after activity blockade was strongly diminished in Rab3A^−/−^ neurons. In the Rab3A^*Ebd/Ebd*^ data set, mEPSC amplitudes from untreated cultures were significantly larger than those of untreated cultures from wild type mice, as can be seen comparing the CON values in Figures 2E and F (CON, Rab3A^+/+^, 11.0 ± 0.7 pA, vs. CON, Rab3A^*Ebd/Ebd*^, 15.1 ± 1.0 pA, p = 0.0027, Kruskal-Wallis test). The increase in mEPSC amplitude in cultures from Rab3A^*Ebd/Ebd*^ mice is consistent with the increase in mEPC amplitude we observed at the Rab3A^*Ebd/Ebd*^ NMJ (Wang et al., 2011). Similar increases in mEPSC amplitude at baseline, combined with a failure to increase further after activity blockade, were noted in cultures prepared from mECP2, AKAP, Homer1a, and Arc/Arg3.1 deletion mice (Shepherd et al., 2006; Hu et al., 2010; Xu and Pozzo-Miller, 2017; Sanderson et al., 2018) as well as in cultures treated with ubiquitin proteasome inhibitors (Jakawich et al., 2010a) or microRNA 186–5p (Silva et al., 2019), suggesting the possibility of a generalized phenomenon in which increased mEPSC amplitude is induced when homeostatic regulation is disrupted. Since we did not observe such an increase in mEPSC amplitude at baseline in cultures from Rab3A^−/−^ mice, it remains a possibility that the point mutant may bind to novel partners, causing activities that would not be either facilitated or inhibited by Rab3A. Still, it is a strong coincidence that the novel activity of the mutant Earlybird Rab3A would affect mEPSC amplitude, the same characteristic that is modulated by activity blockade in a Rab3A dependent manner. Finally, although we cannot rule out that once mEPSC amplitude is increased at baseline, it is not possible for homeostatic plasticity to increase mEPSC amplitude further due to a non-specific ceiling effect, the simplest interpretation is that the presence of the mutant protein mimics the condition of activity blockade.

The small GTPase Rab3A is generally thought to function presynaptically to regulate synaptic vesicle trafficking, possibly in an activity-dependent manner (Castillo et al., 1997; Lonart et al., 1998; Leenders et al., 2001; Schluter et al., 2006; Coleman and Bykhovskaia, 2009; Tian et al., 2012). In contrast, homeostatic plasticity of mEPSC amplitude has been attributed to an increase in postsynaptic receptors on the surface of the dendrite (O’Brien et al., 1998; Turrigiano et al., 1998). Is Rab3A required for the increase in surface AMPA-type glutamate receptors that has been confirmed by multiple studies (see Introduction)? At the NMJ in vivo we could find no evidence for an increase in AChRs after TTX block of the sciatic nerve in vivo (Wang et al., 2005). The type of AMPA receptor that has been shown to be increased, calcium-permeable GluA1-containing AMPA receptor or calcium-impermeable GluA2-containing AMPA receptor, appears to depend on the experimental manipulation. For example, block of activity by APV (NMDA antagonist) combined with TTX (inhibitor of Na^+^ channels) induces an increase in mEPSC amplitude in dissociated hippocampal cultures that is completely reversed by acute application of the Ca^2+^-permeable receptor-specific inhibitor NASPM (Sutton et al., 2006), indicating the entirety of the homeostatic effect is due to an increase in homomeric Ca^2+^-permeable AMPA receptors, likely GluA1, although GluA3 and GluA4 are also Ca^2+^-permeable (Hollmann et al., 1991; Burnashev et al., 1992). Heteromeric receptors of any of these subunits in combination with GluA2 would have rendered the receptors Ca^2+^-impermeable and no longer sensitive to NASPM (Blaschke et al., 1993; Bochet et al., 1994; Jonas and Burnashev, 1995). In contrast, the increase in mEPSC amplitude in mouse hippocampal slice cultures induced by TTX alone was not affected by another Ca^2+^-permeable AMPA receptor inhibitor, philanthotoxin, suggesting mediation by either GluA2-containing AMPA receptors or a presynaptic effect on quantal size (Soden and Chen, 2010); see also, (Dubes et al., 2022).

**Figure 3.**
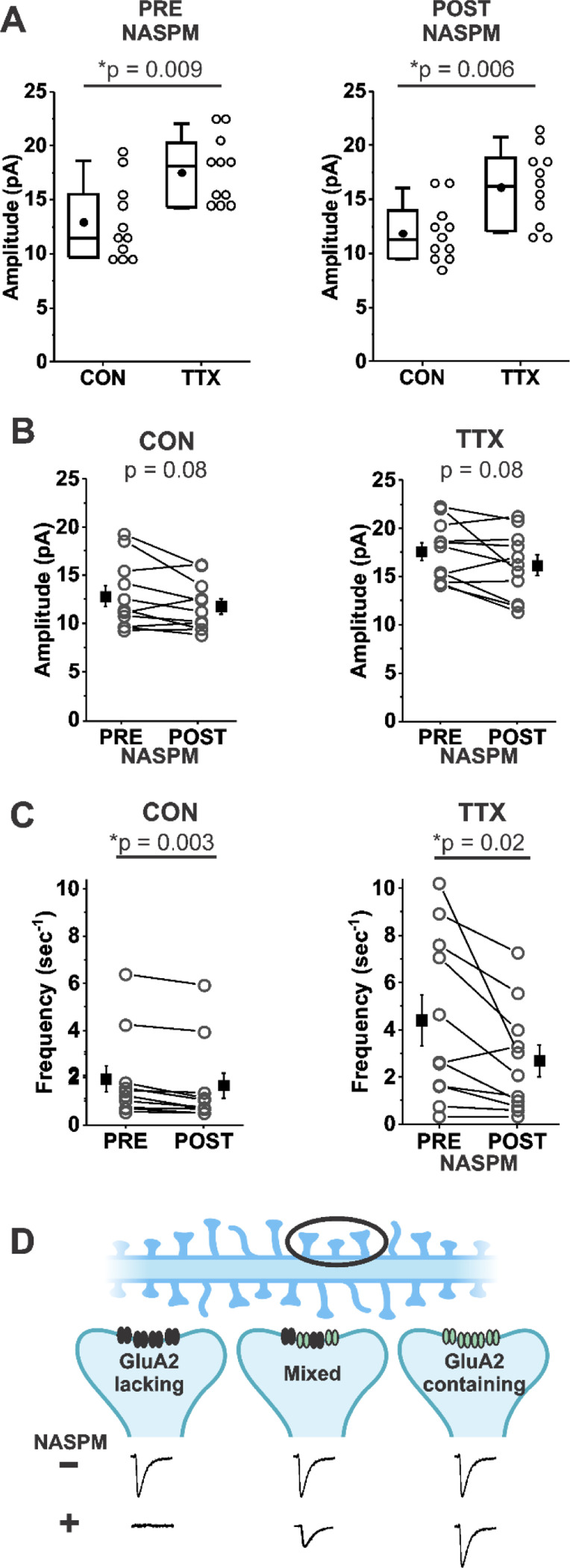
Homeostatic plasticity of mEPSC amplitudes in mouse cortical cultures treated with TTX for 48 hr was not affected by acute inhibition of Ca^2+^ permeable AMPA receptors by NASPM (20 μm). (A) Box plot comparison of the TTX effect on mEPSC amplitudes in the same pyramidal neurons before and after application of 20 μM NASPM. (N = 11 cells from 3 cultures; Pre-NASPM, CON: 12.9 ± 1.1 pA; TTX: 17.5 ± 0.9 pA; post-NASPM, CON: 11.9 ± 0.8 pA; TTX: 16.1 ± 1.0 pA). (B) Line series plot of mEPSC amplitudes before and after acute perfusion with 20 μM NASPM for untreated and TTX-treated pyramidal neurons; same cells as in (A); CON, pre-NASPM: 12.9 ± 1.1 pA; post-NASPM: 11.9 ± 0.8 pA; TTX, pre-NASPM: 17.5 ± 0.9 pA; post-NASPM: 16.1 ± 0.1 pA. (C) Line series plot of mEPSC frequency before and after acute perfusion with 20 μM NASPM for untreated and TTX-treated pyramidal neurons; same cells as in (A); CON, pre-NAPSM: 1.84 ± 0.55 sec^−1^; post-NASPM; 1.56 ± 0.53 sec^−1^; TTX, pre-NASPM: 4.40 ± 1.06 sec^−1^; post-NASPM, 2.68 ± 0.68 sec^-1^. (D) A proposed mechanism for why NASPM had a robust effect on frequency without greatly affecting amplitude. A dendrite with spines (Top) is expanded on three postsynaptic sites (Middle) to show possible types of AMPA receptor distributions: Left, at a site comprised only of Ca^2+^-permeable AMPA receptors (NASPM-sensitive, GluA2-lacking receptors (black)), NASPM would completely inhibit the mEPSC, causing a decrease in frequency to be measured in the overall population; Right, at a site comprised only of Ca^2+^-impermeable AMPA receptors (NASPM-insensitive, GluA2-containing receptors (green)), NASPM would have no effect on mEPSC amplitude; Middle, at a site comprised of a mix of Ca^2+^-permeable and Ca^2+^-impermeable AMPA receptors, NASPM would partially inhibit the mEPSC. Diagram produced in BioRender.com (2024). Box plot parameters: box ends, 25th and 75th percentiles; whiskers, 10th and 90th percentiles; open circles represent means from individual cells; line, median; dot, mean. p values (shown on the graphs) are from Kruskal-Wallis test. Line series plot p values are from student’s paired t test. For all p-values, * with underline indicates significance with p < 0.05.

Because we used TTX alone to block network activity, we expected that NASPM would not reverse the TTX-induced increase in mEPSC amplitude in our mouse cortical cultures, and we found that this was indeed the case. Figure 3A shows that the TTX-induced increase in mean mEPSC amplitude was nearly identical in a set of 11 CON and 11 TTX-treated cells before and after NASPM treatment (before NASPM, CON 12.9 ± 3.5 pA; TTX, 17.5 ± 3.1 pA; after NASPM, CON 11.9 ± 2.6 pA; TTX 16.1 ± 3.5 pA). This result indicates that the TTX-induced increase in mEPSC amplitude does not depend on Ca^2+^-permeable receptors, since the effect of TTX clearly remained when their presence was removed by acute NASPM application. We do not think that there was any technical issue with the NASPM application, because overall, mEPSC amplitudes were reduced modestly in both untreated and TTX-treated cultures (Figure 3B, CON, before NASPM, 12.9 ± 3.5 pA; after, 11.9 ± 2.6 pA; TTX, before NASPM, 17.5 ± 3.1 pA; after, 16.1 ± 3.5 pA). Furthermore, NASPM consistently reduced mEPSC frequency (Figure 3C, CON, before NASPM, 1.84 ± 0.55 sec^−1^; after NASPM, 1.56 ± 0.53 sec^−1^; TTX, before NASPM, 4.40 ± 3.51 sec^−1^; after NASPM, 2.68 ± 2.25 sec^−1^). Similar to the data in Figure 1, frequency is significantly increased after TTX treatment (p = 0.04), but this difference is no longer significant after NASPM application (p = 0.20). A loss in frequency after acute application of NASPM suggests there are synaptic sites that express only homomers of Ca^2+^-permeable receptors; mEPSCs from these sites would be expected to be completely blocked by NASPM (see cartoon description in Figure 3D, Left). The magnitude of the frequency reduction following acute NASPM appears to be greater after TTX treatment, suggesting that loss of activity promotes the establishment of synaptic sites that contain only Ca^2+^-permeable receptors. However, these new sites do not appear to contribute to the increase in mEPSC amplitude (Figure 3A). The lack of effect of NASPM also rules out the contribution of Ca^2+^-permeable forms of Kainate receptors GluA5 and GluA6 (Koike et al., 1997; Sun et al., 2009).

**Figure 4.**
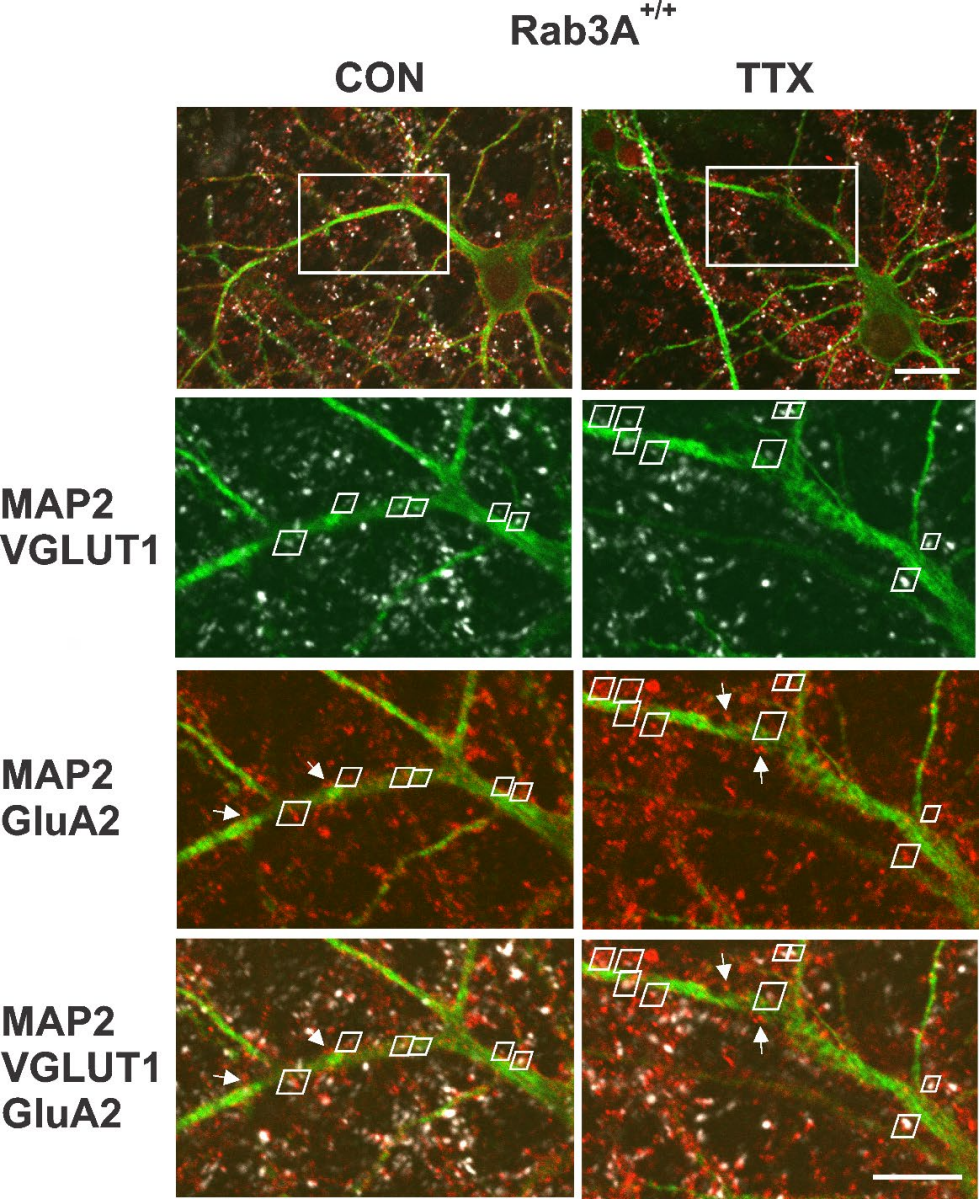
Identification of synaptic GluA2 receptor immunofluorescence on primary dendrites of pyramidal neurons in high density mouse cortical co-cultures prepared from Rab3A^+/+^ mice. (Top) Non-zoomed single confocal sections collected with a 60X oil immersion objective of recognizably pyramidal-shaped neurons, presumed to be excitatory neurons, selected for synaptic GluA2 analysis. A neuron was selected from an untreated coverslip (CON, Left) and a TTX-treated coverslip (TTX, Right) from the same culture prepared from Rab3A^+/+^ animals (=Rab3A^+/+^ Culture #2 in Figure 6). The white rectangular boxes indicate 5X zoomed areas shown in the images below. Note that the depth of the single confocal section of the non-zoomed neuron image is not at the same depth as the confocal section in zoomed dendritic image, so some features are not visible in both images. Scale bar, 20 μm. (Bottom) 5X zoom single confocal sections selected for demonstration purposes because they had an unusually high number of identified synaptic pairs along the primary dendrite contained within a single confocal section. Synaptic pairs, highlighted with white trapezoids, were identified based on close proximity of GluA2 (red) and VGLUT1 (white) immunofluorescence, apposed to the MAP2 immunofluorescent primary dendrite (green). Some apparent synaptic pairs are not highlighted with a white trapezoid because there was a different confocal section in which the two immunofluorescent sites were maximally bright. There were GluA2 positive clusters located on the primary dendrites that were not apposed to VGLUTl-positive terminals (4 of these non-synaptic GluA2 clusters are highlighted with white arrows in the MAP2-GluA2 and MAP2-GluA2-VGLUT1 panels). Scale bar, 10 μm.

Having established that homomeric Ca^2+^-permeable receptors are not contributing to the homeostatic increase in mEPSC amplitude, we turned to immunohistochemistry and confocal imaging to assess whether GluA2 receptor expression, which will identify either GluA2 homomers or GluA1-GluA2 heteromers, was increased in our wild type mouse cortical cultures following 48 hr treatment with TTX. Since mEPSCs necessarily report synaptic levels of receptors, we used VGLUT1-immunofluorescence to identify synapses on pyramidal primary apical dendrites labeled with MAP-2 immunofluorescence. We focused on the primary dendrite of pyramidal neurons as a way to reduce variability that might arise from being at widely ranging distances from the cell body, or, from inadvertently sampling dendritic regions arising from other neurons, possibly inhibitory neurons. Figure 4 (Top) shows two pyramidal neurons, one from an untreated culture on the left, and one from a TTX-treated culture on the right, prepared from Rab3A^+/+^ mice. To measure characteristics of synaptically located GluA2 receptor clusters, we zoomed in on the primary dendrite (region in white rectangle). The zoomed regions for single confocal sections of the selected area are shown below, and contain pairs of VGLUT1- and GluA2-immunofluorescent clusters indicated by white trapezoidal frames. A dendrite typically required ~10 confocal sections to be fully captured, and the total number of synaptic pairs for all the sections imaged on a dendrite was usually < 20, so this is an atypically high number of pairs within a single section; these particular dendrites and sections were selected for illustration purposes. In addition to the synaptic pairs, we observed many GluA2-immunofluorescent clusters not associated with VGLUT1 immunofluorescence (a few are indicated with white arrows), most probably extrasynaptic receptors. There were also GluA2-positive clusters present outside of MAP2-positive dendrites, which may be located on astrocytes (Fan et al., 1999). Finally, GluA2 immunofluorescent clusters close to VGLUT1 immunofluorescence but not located along any apparent MAP2-positive neurites suggests the presence of axon-axonal contacts, although VGLUT1 has also been detected in astrocytes (Ormel et al., 2012). Only sites that contained both VGLUT1 and GluA2 immunofluorescence close to the primary MAP2-postive dendrite or a secondary branch along this primary dendrite were selected for analyses. In the experiment from which these images were collected, we analyzed the distance from the cell body for each synapse. The average distance from the cell body, for dendrites from the untreated cell, was 38.5 ± 2.8 μm; for the TTX-treated cell, it was 42.4 ± 3.2 μm (p = 0.35, Kruskal-Wallis test). Since the greatest distance from the cell body is set by the outer limit of the zoom window, and that window was placed adjacent to the cell body, distances in other experiments would be in this same range.

**Figure 5.**
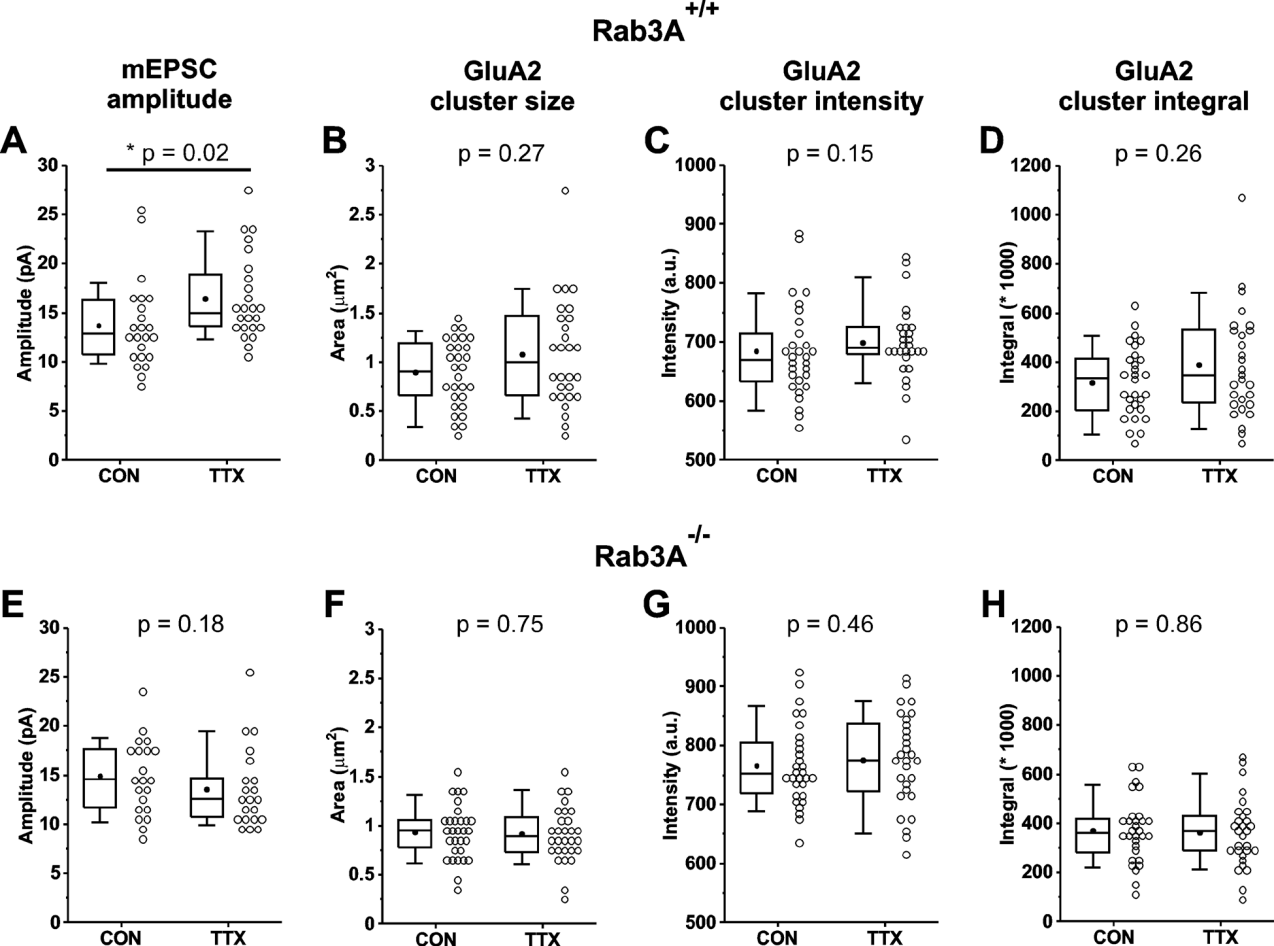
Comparison of mEPSC amplitude and GluA2 receptor cluster data, pooled from 3 cortical co-cultures prepared from Rab3A^+/+^ mice and 3 co-cultures prepared from Rab3A^−/−^ mice. (A) Mean mEPSC amplitudes recorded in pyramidal neurons from untreated (CON) and TTX-treated coverslips (TTX) of co-cultures prepared from Rab3A^+/+^ mice (CON, N = 23 cells, 13.7 ± 0.9 pA; TTX, N = 24 cells, 16.4 ± 0.9 pA; from 3 cultures). (B) Mean sizes of synaptic GluA2 receptor clusters, identified by proximity to VGLUT1-positive terminals, on primary dendrites of pyramidal neurons of untreated and TTX-treated coverslips from the same cultures as in (A) (CON, N = 29 dendrites, 0.90 ± 0.06 μm^2^; TTX, N = 28 dendrites, 1.08 ± 0.10 μm^2^). (C) Mean intensities of the same GluA2 receptor clusters as in (B) (CON, 684 ± 15 A.U.; TTX, 699 ± 12 A.U.). (D) Mean integrals of the same GluA2 receptor clusters as in (B). (CON, 317,898 ± 26,726 A.U.; TTX, 389,487 ± 41,465 A.U.). (E) Mean mEPSC amplitudes in pyramidal neurons from untreated and TTX-treated coverslips of mouse cortical co-cultures from Rab3A^−/−^ mice (CON, N = 21 cells, 14.9 ± 0.8 pA; TTX, N = 21 cells, 13.5 ± 0.9 pA; from 3 cultures). (F) Mean sizes of GluA2 receptor clusters, identified by proximity to VGLUT1-positive terminals, on primary dendrites of pyramidal neurons of untreated and TTX-treated coverslips from the same cultures as in (E). (CON, N = 30 dendrites, 0.93 ± 0.05 μm^2^; TTX, N = 30 dendrites, 0.92 ± 0.05 μm^2^). (G) Mean intensities of the same GluA2 receptor clusters as in (F). (CON, 766 ± 12 A.U.; TTX, 776 ± 78 A.U.). (H) Mean integrals of the same GluA2 receptor clusters as in (F). (CON, 369,436 ± 23,439 A.U.; TTX, 366,389 ± 25,049 A.U.). Box plot parameters: box ends, 25th and 75th percentiles; whiskers, 10 and 90 percentiles; open circles indicate means of individual cells; line, median; dot, mean. p values (shown on the graphs) were determined with Kruskal-Wallis test. For all p-values, * with underline indicates significance with p < 0.05.

Variability in the magnitude of the homeostatic response from culture to culture is averaged out in physiological experiments by the pooling of data from many cells across many cultures. To reduce the necessity for many cultures, we chose to pair experiments in the same cultures by recording mEPSCs from one set of coverslips, and processing another set of coverslips from the same culture for immunofluorescence. We completed this matched paradigm of physiology and immunofluorescence, which to our knowledge has never before been done, for 3 cultures prepared from Rab3A^+/+^ mice and 3 cultures prepared from Rab3A^−/−^ mice. We present the results for mEPSC data and imaging data pooled across the 3 experiments in Figure 5, with the data summarized in Table 1. Levels of GluA2 immunofluorescence at synaptic sites were quantified by the size of the GluA2-immunofluorescent receptor cluster, the average intensity value, and the integral of intensity values across the area of the cluster. In the data pooled from the 3 matched experiments, neurons from Rab3A^+/+^ cultures showed a significant increase in mean mEPSC amplitudes following activity blockade (Figure 5A; Table 1). This result indicates that the homeostatic response averaged across the 3 cultures was very similar to, but slightly smaller than, that of the data set presented in Figure 1. The means for size, intensity and integral of GluA2 immunofluorescent receptor clusters showed trends to higher values after activity blockade, but the differences did not reach statistical significance (Figure 5B size; Figure 5C intensity; Figure 5D integral; Table 1). These results support previous studies demonstrating an increase in AMPA receptors following activity blockade, with the magnitude of the changes in GluA2 receptor cluster size and integral matching that of mEPSC amplitude, at around 20% (Table 1), but not achieving the same level of statistical significance.

As expected, mean mEPSC amplitude was not increased following activity blockade in the data pooled from the new set of 3 Rab3A^−/−^ cultures (Figure 5E). For immunofluorescence results from the same Rab3A^−/−^ cultures, no trend towards higher values was apparent for GluA2 receptor cluster characteristics (Figure 5F size; Figure 5G intensity; Figure 5H integral; Table 1). Taken together, these results indicate that the modest increases in GluA2 receptor cluster size and integral following activity blockade observed in wild-type cultures do not occur in the absence of Rab3A. These results suggest the surprising finding that the presynaptic vesicle protein Rab3A is involved in the regulation of postsynaptic AMPA receptors during homeostatic plasticity.

**Figure 6.**
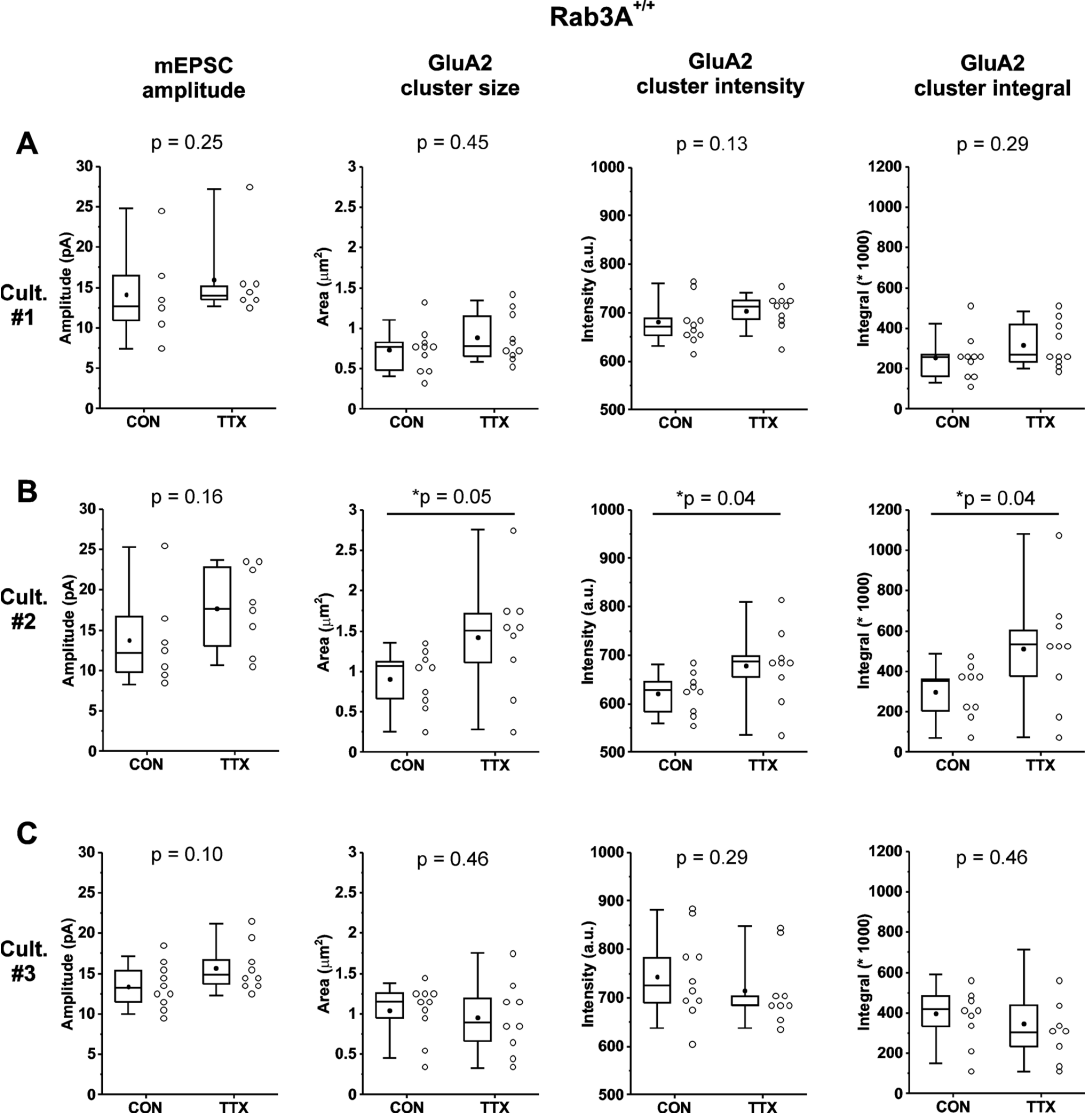
In cultures prepared from Rab3A^+/+^ mice, increases in mEPSC amplitudes following TTX treatment were not always matched by increases in GluA2 receptor cluster characteristics. (A) Culture #1: mean mEPSC amplitudes for pyramidal neurons from untreated coverslips (CON) and TTX-treated coverslips (TTX) (CON, N = 6, 14.2 ± 2.2 pA; TTX, N = 6, 15.9 ± 1.9 pA), compared to mean sizes, intensities, and integrals of synaptic GluA2 receptor clusters in images taken of dendrites from other coverslips in the same culture (CON, N = 10, TTX, N = 9; size, CON, 0.73 ± 0.09 μm^2^; TTX, 0.89 ± 0.09 μm^2^; intensity, CON, 682 ± 15 A.U.; TTX, 704 ± 11 A.U.; integral, CON, 256,364 ± 35,242 A.U.; TTX, 316,647 ± 35,077 A.U.). (B) Culture #2: mean mEPSC amplitudes for pyramidal neurons from untreated and TTX-treated coverslips (CON, N = 7, 13.8 ± 2.4 pA; TTX, N = 8, 17.7 ± 1.8 pA), compared to mean sizes, intensities, and integrals of synaptic GluA2 receptor clusters in images taken of dendrites from other coverslips from the same culture (CON, N = 9, TTX, N = 9; size, CON, 0.91 ± 0.12 μm^2^; TTX, 1.42 ± 0.24 μm^2^; intensity, CON, 621 ± 14 A.U.; TTX, 679 ± 26 A.U.; integral, CON, 296,300 ± 44,108 A.U.; TTX, 512,336 ± 96,892 A.U.). (C) Culture #3: mean mEPSC amplitudes for pyramidal neurons from untreated coverslips and TTX-treated coverslips (CON, N = 10, 13.4 ± 0.8 pA; TTX, N = 9, 15.7 ± 1.0 pA,) compared to mean sizes, intensities, and integrals of synaptic GluA2 receptor clusters in images taken from other coverslips from the same culture. (CON, N = 10, TTX, N = 9; size, CON, 1.05 ± 0.11 μm^2^; TTX, 0.95 ± 0.15 μm^2^; intensity, CON, 744 ± 28 A.U.; TTX, 714 ± 25 A.U.; integral, CON, 398,870 ± 49,500 A.U.; TTX, 347,571 ± 65,454 A.U.). Cultures were fixed and processed for immunofluorescence on the same day as mEPSC recordings, and imaged within 1 week. Box plot parameters: box ends, 25th and 75th percentiles; whiskers, 10th and 90th percentiles; open circles represent means from individual cells; line, median; dot, mean. p values (shown on the graphs) are from Kruskal-Wallis test. p-values denoted with * and underline indicates significance of p < 0.05.

As noted above, the homeostatic increase in mEPSC amplitude in cultures from Rab3A^+/+^ mice reached significance whereas that of receptor levels did not. To determine where this disparity was coming from, we compared mEPSC amplitude and GluA2 receptor data within individual experiments. In order to evaluate whether a particular culture displayed homeostatic plasticity of mEPSC amplitude, we set a minimum requirement of recording from 6 cells per condition, meaning a successful experiment required at least 12 usable recordings in one day (6 CON and 6 TTX; i.e. low holding current, stable baseline, low noise, etc.). Ultimately, our sample size ranged from 6 to 10 cells per condition per culture. Figures 6A-C show the box plots for mEPSC amplitudes, and GluA2 receptor cluster size, intensity and integrals, for 3 individual Rab3A^+/+^ cultures. With these small sample sizes, the differences between untreated and TTX-treated cells were not statistically significant for mEPSC amplitudes in any of the 3 cultures, but an upward trend occurred in every case, ranging from 12 to 28% (Table 1). Second, in two of the three Rab3A^+/+^ experiments (Cultures #1 and #2) GluA2 receptor cluster size increased 22 and 56%, respectively, significantly so for Culture #2 (Table 1). However, in one experiment (Culture #3), GluA2 receptor cluster size showed a downward trend of 9.5%. Because we have 10 CON mean mEPSC amplitudes and 9 TTX mean mEPSC amplitudes that indicate this particular culture very likely has a normal homeostatic increase in mEPSC amplitude induced by activity blockade, the lack of increase in GluA2 receptor cluster characteristics suggests that mEPSC amplitudes can increase in the absence of an increase in receptors. Furthermore, Culture #3’s opposite results from Cultures #1 and #2, explain why in the pooled data set, the increase in GluA2 receptor characteristics after TTX treatment do not reach statistical significance.

**Figure 7.**
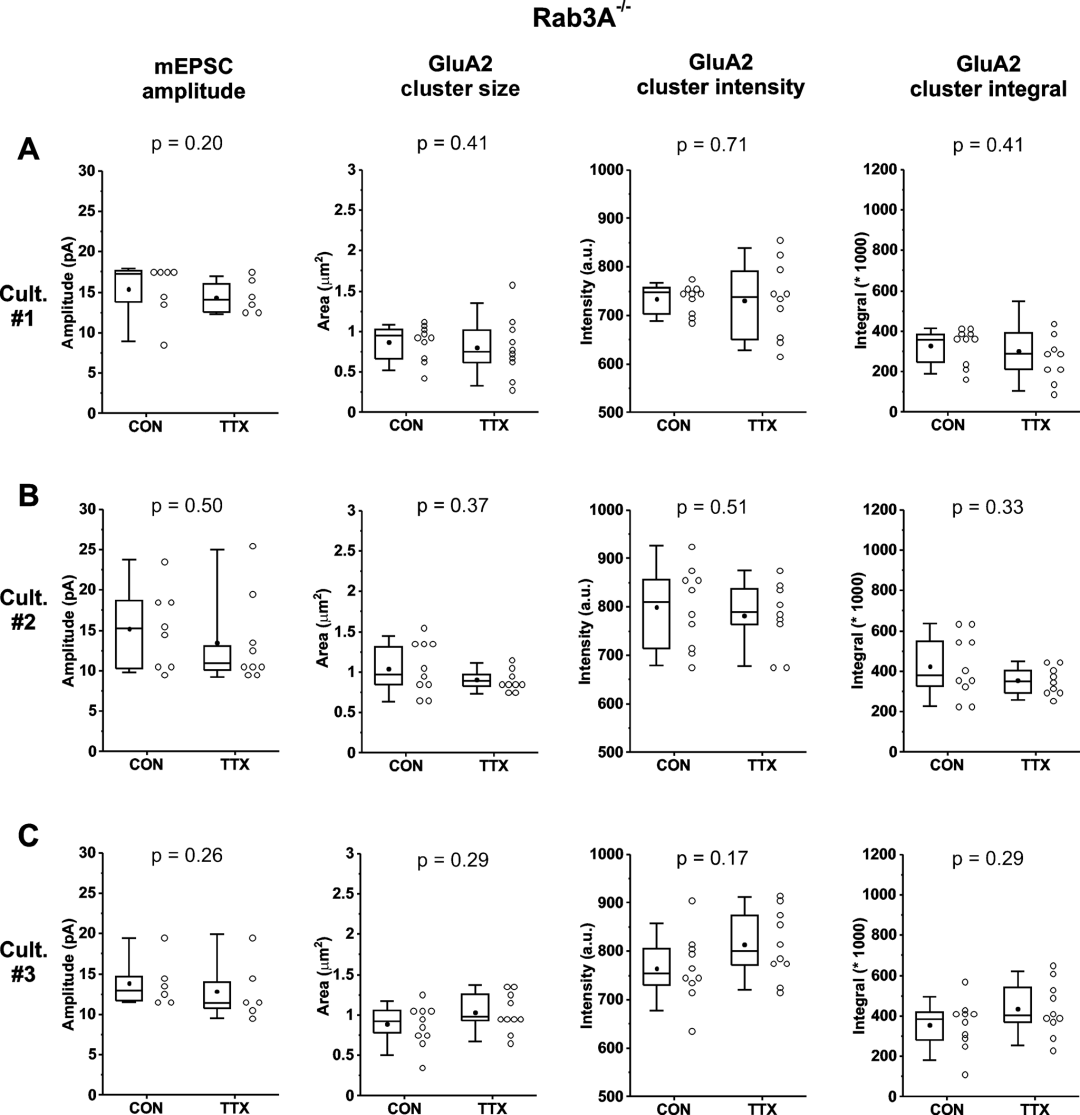
In cultures prepared from Rab3A^−/−^ mice, a lack of effect of TTX treatment in mEPSC amplitudes was not always matched by a lack of effect in synaptic GluA2 receptor cluster characteristics. (A) Culture #1: mean mEPSC amplitudes for pyramidal neurons from untreated coverslips (CON) and TTX-treated coverslips (TTX) (CON, N = 7, 15.4 ± 1.2 pA; TTX, N = 6, 14.4 ± 0.8 pA), compared to mean sizes, intensities, and integrals of synaptic GluA2 receptor clusters in images taken of dendrites from other coverslips in the same culture (CON, N = 10, TTX, N = 10; size, CON, 0.87 ± 0.07 μm^2^; TTX, 0.80 ± 0.12 μm^2^; intensity, CON, 735 ± 10 A.U.; TTX, 731 ± 25 A.U.; integral, CON, 328,477 ± 28,250; TTX, 302,594 ± 53,052 A.U.). (B) Culture #2: mean mEPSC amplitudes for pyramidal neurons from untreated and TTX-treated coverslips (CON, N = 8, 15.2 ± 1.8 pA; TTX, N = 9, 13.5 ± 1.8 pA), compared to mean sizes, intensities, and integrals of synaptic GluA2 receptor clusters in images taken of dendrites from other coverslips in the same culture (CON, N = 10, TTX, N = 10; size, CON, 1.05 ± 0.10 μm^2^; TTX, 0.93 ± 0.05 μm^2^; intensity, CON, 800 ± 26 A.U.; TTX, 781 ± 21 A.U.; integral, CON, 425,552 ± 49,486 A.U.; TTX, 361,740 ± 21,963 A.U.). (C) Culture #3: mean mEPSC amplitudes for pyramidal neurons from untreated and TTX-treated coverslips (CON, N = 6, 13.9 ± 1.2 pA; TTX, N = 6, 12.9 ± 1.5 pA) compared to mean sizes, intensities, and integrals of synaptic GluA2 receptor clusters in images taken of dendrites from other coverslips in the same culture (CON, N = 10, TTX, N = 10; size, CON, 0.89 ± 0.08 μm^2^; TTX, 1.03 ± 0.08 μm^2^; intensity, CON, 765 ± 22 A.U.; TTX, 814 ± 23 A.U.; integral, CON, 354,279 ± 38,755 A.U.; TTX, 434,833 ± 42,350 A.U.). Cultures were fixed and processed for immunofluorescence on the same day as mEPSC recordings, and imaged within 1 week. Box plot parameters: box ends, 25th and 75th percentiles; whiskers, 10th and 90th percentiles; open circles represent means from individual cells; line, median; dot, mean. p values (shown on the graphs) are from Kruskal-Wallis test.

Figures 7A-C show the box plots for mEPSC amplitudes, and GluA2 receptor cluster size, intensity and integrals, for 3 individual Rab3A^−/−^ cultures. Here, mEPSC amplitudes showed a downward trend in all 3 cultures, as did GluA2 receptor characteristics for two of the three cultures. But we found another obvious mismatch between the mEPSC amplitude data and the GluA2 receptor cluster data for one of the Rab3A^−/−^ cultures. For Culture #3 (Figure 7C), the box plot of mEPSC mean amplitudes showed a downward trend after TTX treatment, decreasing by 7% (Table 1), whereas GluA2 receptor cluster size, intensity and integral all showed upward trends, the increase ranging from 6 to 23% (Table 1). This result may suggest that the disruption of the homeostatic increase in receptors in the absence of Rab3A is not absolute. It also indicates that synaptic GluA2 receptor levels can increase without an accompanying increase in mEPSC amplitudes.

In summary, our matched mEPSC and receptor cluster results indicate that: 1. activity blockade resulted in an increase in mEPSC amplitudes usually accompanied by an increase in synaptic GluA2 receptors; 2. loss of Rab3A disrupted both the increase in mEPSC amplitudes and the increase in GluA2 receptor levels; and 3. The differences between homeostatic responses of mEPSC amplitudes and the GluA2 cluster sizes within the same cultures suggest there may be other factors that contribute to mEPSC amplitude.

Having used VGLUT1 immunofluorescence to mark synaptic sites, we could also ask whether there was a presynaptic effect of activity blockade on the size, intensity or integral of the VGLUT1 signal at sites apposed to the previously examined GluA2 postsynaptic sites. In the pooled data from cultures prepared from Rab3A^+/+^ mice, we found no change in the size of the VGLUT1-positive regions, and trends towards reduced intensity and integral (Table 2). These data do not support the idea that activity blockade caused an increase in the amount of VGLUT1 per vesicle, although we cannot rule out that a simultaneous reduction in the number of vesicles obscured the effect, since we did not have an independent label for synaptic vesicles. In cultures prepared from Rab3A^−/−^ mice, the size, intensity and integral of the VGLUT1-positive regions showed upward trends (Table 2), suggesting the possibility that the modest decreases observed for cultures prepared from wild type mice depend on Rab3A, but it is difficult to make any conclusions when the p values are far from reaching significance. Similarly to the GluA2 receptor cluster data, the likely reason that the pooled data effects did not reach significance is because the effects were not consistent across all three cultures, with one Rab3A^+/+^ culture showing an upward trend instead of a downward trend, and one Rab3A^−/−^ culture showing a downward trend instead of an upward trend (Table 2). It should be noted that there was no consistent inverse pattern for GluA2 vs. VGLUT1. While in cultures prepared from Rab3A^−/−^ mice, when GluA2 characteristics trended downward, VGLUT1 characteristics trended upward, this pattern did not hold for cultures prepared from Rab3A^+/+^ mice. In Rab3A^+/+^ Culture #2, GluA2 showed significant increases, and VGLUT1 showed essentially no change. In Rab3A^+/+^ Culture #3, both GluA2 and VGLUT1 trended downwards. Taken together, the GluA2 and VGLUT1 immunofluorescence data indicate that GluA2 receptors likely contribute to the increase in mEPSC amplitudes after activity blockade in Rab3A^+/+^ cultures, and the disruption of that increase in Rab3A^−/−^ cultures, but VGLUT1 levels change in the opposite direction from mEPSC amplitudes. Interestingly, VGLUT1 levels are strongly affected by loss of Rab3A, with a highly significant decrease in integral in untreated cultures from Rab3A^−/−^ mice, compared to untreated cultures from Rab3A^+/+^ mice (size, p = 0.019; intensity, p = 0.004; integral, p = 4.6 * 10^−4^; Table 2).

**Figure 8.**
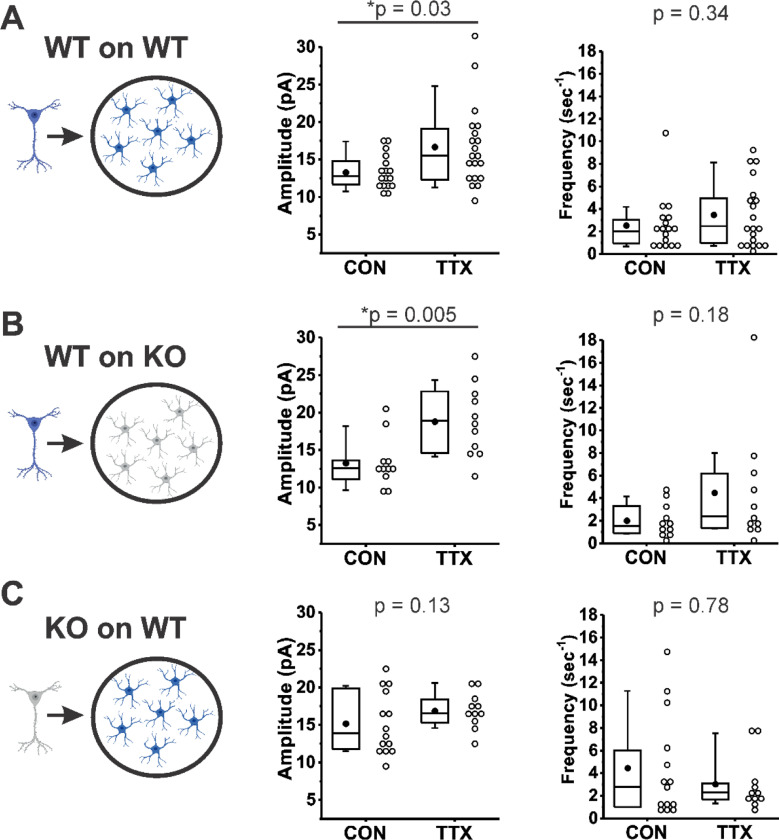
Rab3A in neurons, not astrocytes, was required for full TTX-induced homeostatic plasticity. (A)-(C), mEPSC amplitude (Middle) and frequency (Right) data from dissociated cortical neurons plated on an astrocyte feeder layer, each prepared separately from the type of mice depicted in the schema (Left): (A) Neurons from Rab3A^+/+^ mice plated on astrocytes from Rab3A^+/+^ mice. Box plots for mEPSC amplitudes (CON, N = 17 cells, 13.3 ± 0.5 pA; TTX, N = 20 cells, 16.7 ± 1.2 pA) and mEPSC frequency in the same recordings (mean, CON, 2.54 ± 0.57 sec^−1^; TTX, 3.48 ± 0.64 sec^−1^); from 4 cultures. (B) Neurons from Rab3A^+/+^ mice plated on astrocytes from Rab3A^−/−^ mice. Box plots for average mEPSC amplitude (CON, N = 11 cells, 13.3 ± 1.0 pA; TTX, N = 11 cells, 18.8 ± 1.4 pA) and average mEPSC frequency in the same recordings (CON, 2.01 ± 0.41 sec^−1^; TTX, 4.47 ± 1.53 sec^−1^); from 2 cultures. (C) Neurons from Rab3A^−/−^ neurons plated on astrocytes from Rab3A^+/+^ mice. Box plots for average mEPSC amplitude (CON, N = 14 cells, 15.2 ± 1.1 pA; TTX, N = 11 cells, 16.9 ± 0.7 pA) and mEPSC frequency in the same recordings (CON, 4.47 ± 1.21 sec^−1^; TTX, 3.02 ± 0.70 sec^−1^); from 3 cultures. Box plot parameters: box ends, 25th and 75th percentiles; whiskers, 10th and 90th percentiles; open circles represent means from individual cells; line, median; dot, mean. p values (shown on the graphs) are from Kruskal-Wallis test. p-values denoted with * and underline indicates significance of p < 0.05.

We have demonstrated here for the first time that the synaptic vesicle protein Rab3A influences the homeostatic regulation of postsynaptic GluA2 receptors. What are possible ways that Rab3A could exert a postsynaptic action? It has previously been shown that exogenous addition of TNFα to hippocampal cultures causes an increase in surface expression of GluA1 receptors (although not GluA2 receptors), and that the homeostatic increase in mEPSC amplitudes is abolished in cultures prepared from the TNFα deletion mouse (Stellwagen et al., 2005; Stellwagen and Malenka, 2006). Furthermore, neurons from TNFα^+/+^ mice plated on glial feeder layers derived from TNFα^−/−^ mice fail to show the increase in mEPSC amplitude after TTX treatment, indicating that the TNFα inducing the receptor increases following activity blockade comes from the glial cells (Stellwagen and Malenka, 2006). It has recently been shown that the source of TNFα is astrocytes rather than microglia (Heir et al., 2024). Rab3A has been detected in astrocytes (Maienschein et al., 1999; Hong et al., 2016), so to determine whether Rab3A is acting via regulating TNFα release from astrocytes, we performed experiments similar to those of Stellwagen and Malenka (2006) (schema illustrated in Figure 8, Left). It should be noted that under our culturing conditions, the glial feeder layers would be expected to be composed mainly of astrocytes (Heir et al., 2024). We compared the effect of activity blockade on mEPSC amplitudes recorded from cortical neurons from Rab3A^+/+^ mice plated on Rab3A^+/+^ glial feeder layers (Figure 8A); neurons from Rab3A^+/+^ mice plated on Rab3A^−/−^ glia (Figure 8B), and neurons from Rab3A^−/−^ mice plated on Rab3A^+/+^ glia (Figure 8C). If Rab3A is required for TNFα release from glia, then Rab3A^+/+^ neurons plated on Rab3A^−/−^ glia should not show a homeostatic increase in mEPSC amplitude after treatment with TTX, and any cultures with Rab3A^+/+^ glia should have a normal homeostatic response. We found the opposite result: mEPSC amplitudes increased dramatically in cultures where Rab3A was present in neurons (WT on WT, CON, 13.3 ± 0.5 pA, TTX, 16.7 ± 1.2 pA; WT on KO, CON, 13.3 ± 1.0 pA, TTX, 18.8 ± 1.4 pA), but the increase was greatly diminished in cultures where Rab3A was present only in glia (KO on WT, CON, 15.2 ± 1.1 pA, TTX, 16.9 ± 0.7 pA). It is interesting to note that in the glial feeder layer culture, the loss of Rab3A in the neurons may have caused an increase in mEPSC amplitude at baseline, similar to the Earlybird mutant in dissociated cultures, but it is also possible this can be attributed to culture to culture variability. Regarding mEPSC frequency, in cultures with Rab3A^+/+^ neurons, frequency trended upward after activity blockade, whereas in the culture with Rab3A^−/−^ neurons, frequency was already increased, and trended downward after activity blockade.

In summary, in the absence of Rab3A from glia, the homeostatic plasticity of mEPSC amplitude following activity blockade was completely normal, whereas in the absence of Rab3A in neurons, homeostatic plasticity was greatly diminished. This result makes it highly unlikely that Rab3A is required for the release of TNFα, or another factor from glia, that induces a homeostatic upregulation of postsynaptic receptors and thereby increases mEPSC amplitude following TTX treatment. Neuronal Rab3A appears to mediate the homeostatic increase in mEPSC amplitude following activity blockade.

## Discussion

We found that homeostatic synaptic plasticity of mEPSC amplitude in dissociated mixed cultures of mouse cortical neurons and glia behaved remarkably similar to the mouse NMJ in response to loss of Rab3A function: in cultures from Rab3A^−/−^ mice, the increase in mEPSC amplitudes following prolonged network silencing by TTX was strongly diminished, and in cultures from Rab3A^*Ebd/Ebd*^ mice, basal mEPSC amplitude was increased compared to that of wild-type cultures and was not further modified by TTX treatment. These results suggest that normal function of the synaptic vesicle protein Rab3A is required for the homeostatic increase of mEPSC amplitude in cortical cultures. The effect of TTX on mEPSC amplitude was not due to increases in Ca^2+^-permeable homomers because acute NASPM application was unable to reverse the increase in amplitude after TTX treatment. We used imaging of immunofluorescence to demonstrate a strong trend towards increased GluA2 levels at synapses in activity-blocked cultures from Rab3A^+/+^ mice that was no longer apparent in the absence of Rab3A, suggesting the surprising idea that this protein, previously implicated primarily in the regulation of calcium-dependent vesicle fusion, is also important for the regulation of surface expression of postsynaptic receptors. We found a significant increase in mEPSC frequency after activity blockade that was no longer significant in the absence of Rab3A, which was less surprising as frequency is known to depend on presynaptic factors. At the neuromuscular junction, higher mEPC frequencies that occur in 0.5 mM extracellular calcium and above were significantly reduced in the Rab3A deletion mouse (Coleman et al., 2007). On the other hand, the increase in frequency after TTX treatment is also strongly diminished after acute application of NASPM, suggesting there may be an increase in the number of synapses with only Ca^2+^-permeable homomers. It remains to be determined if this effect is Rab3A-dependent or if there are two distinct mechanisms contributing to homeostatic plasticity of frequency.

The levels of the glutamate transporter, VGLUT1, were not increased after activity blockade under our experimental conditions. A similar lack of increase in VGLUT1 levels, relative to synapsin in imaging of immunofluorescence, was previously observed in hippocampal cultures treated with NB0X, an AMPA receptor blocker (see supplemental data, (Wilson et al., 2005)). On the other hand, treatment with AP5, an NMDA receptor blocker, was able to induce an increase in VGLUT1 levels relative to synapsin (Wilson et al., 2005), suggesting that this presynaptic response depends on the type of activity manipulation. Another study showed increased VGLUT1 mRNA and protein in western blots in cortical cultures treated with TTX, but only examined VGLUT1 at synapses using imaging following treatment with bicuculline (De Gois et al., 2005). Taken together with our findings, it appears that there is not strong evidence that the amount of VGLUT1 at synapses is a contributing factor to the increase in mEPSC amplitude after activity blockade with TTX.

### Homeostatic plasticity of postsynaptic receptors was less consistent than that of mEPSC amplitude

While we found that the effect of TTX treatment on mEPSC amplitude and GluA2 receptor levels were of very similar magnitude, the effect on GluA2 receptor levels was not statistically significant for the pooled data sets acquired in the same cultures. We determined that this discrepancy was due to one of the three cultures showing a downward trend in GluA2 receptor levels after activity blockade. In light of the extensive literature documenting increases in GluA1 or GluA2 receptors, or both, after activity blockade, the less than convincing increase in GluA2 receptor levels in the current work came as a surprise. Therefore we reviewed what has previously been shown, and were able to identify only 5 studies that examined the effect of activity blockade using TTX alone, reported levels of AMPA receptors specifically localized to synapses, and recorded mEPSCs in the same study, although not in the same cultures (Hou et al., 2008; Ibata et al., 2008; Jakawich et al., 2010a; Xu and Pozzo-Miller, 2017; Dubes et al., 2022). Since our issue is not with the magnitude of the changes being different for receptors and mEPSC amplitudes, but with statistical significance (or consistency) being different between the two methods, we wanted to determine whether the previous studies showed any evidence of greater variability in receptor measurements. Only a single study had comparable sample sizes for receptors and mEPSC amplitudes (either because number of receptor sites is used instead of cells, or because the number of cells differs for the two types of experiments), and the p value was < 0.01 for mEPSC amplitudes (n = 9 and 10 cells for CON and TTX, respectively), but < 0.05 for receptor data (n = 13 and 14 cells for CON and TTX) (Xu and Pozzo-Miller, 2017). This single example is consistent with the idea that the receptor data are more variable than mEPSC data. Even if a difference in consistency could be established in multiple studies, it remains a possibility that the disparity is attributable to the unique limitations of the two types of experiments. Nevertheless, the finding that mEPSC amplitude can increase despite a decrease in receptors suggests receptors may not be the only contributor to the TTX effect on mEPSC amplitude.

### Neuronal, not glial, Rab3A may be required for the homeostatic release of a signaling molecule

Our results support the surprising possibility that the presynaptic vesicle protein Rab3A regulates postsynaptic AMPARs. Astrocytic release of TNFα was shown to mediate the increase in mEPSC amplitudes in prolonged TTX-treated cultures of dissociated hippocampal neurons and cultures of hippocampal slices (Stellwagen and Malenka, 2006; Heir et al., 2024), but we found that loss of Rab3A in glia did not disrupt the increase in mEPSC amplitude after activity blockade. Further, we previously showed that the homeostatic increase in NMJ mEPC amplitude was completely normal in the absence of TNFα (Wang et al., 2011). This leaves neuronal Rab3A as the potential controller of a soluble factor regulating surface expression of receptors.

**Figure 9.**
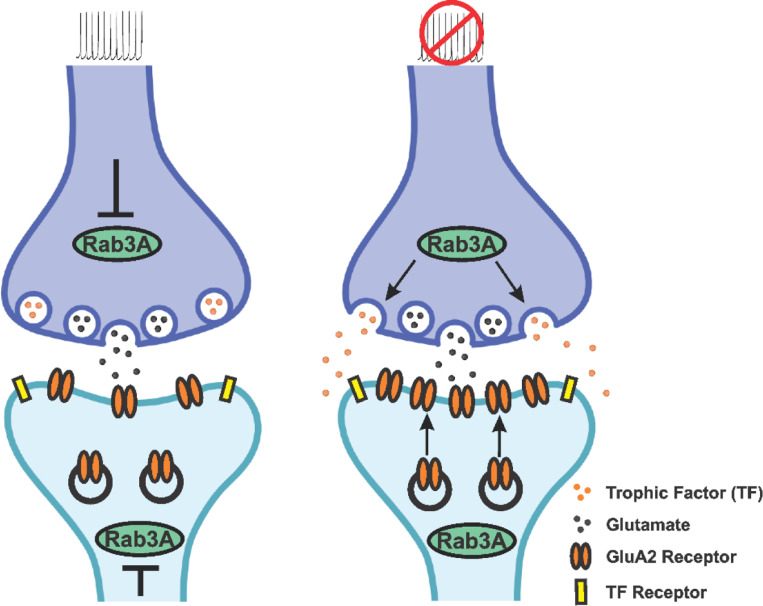
Model depicting the requirement for neuronal Rab3A in the homeostatic increase in synaptic GluA2 receptor levels after treatment with TTX. Under normal activity levels (Left), neither presynaptic or postsynaptic Rab3A is engaged in the regulation of GluA2 receptor levels. After prolonged activity blockade (Right), Rab3A located in the presynaptic terminal promotes the release of a trophic factor (orange dots) that diffuses across the synapse and facilitates transport of postsynaptic vesicles containing GluA2 AMPA receptors (dark orange channels) to the surface membrane. Rab3A located in the postsynaptic dendrite directly promotes fusion of vesicles containing GluA2 AMPA receptors. Fusion of presynaptic vesicles containing glutamate (black dots) causes mEPSCs when glutamate binds to AMPA receptors. Adapted from “Synaptic Cleft” template, by BioRender.com (2024).

One possible signaling molecule being controlled by Rab3A is brain-derived neurotrophic factor (BDNF). Addition of exogenous BDNF to neuronal cultures prevents the increase in mEPSC amplitude following activity blockade (Rutherford et al., 1998; Benevento et al., 2016) but see (Smith-Dijak et al., 2019), and BDNF mRNA is reduced after activity blockade in vitro (Benevento et al., 2016; Miyasaka and Yamamoto, 2021) and in vivo (Castren et al., 1992). Also, after reduction of secreted BDNF in culture media via the BDNF scavengers TrkB-FC or TrkB-IgG, mEPSC amplitudes are increased (Rutherford et al., 1998; Benevento et al., 2016; Smith-Dijak et al., 2019). Reduction of Rab3A in astrocytes decreases astrocytic BDNF release (Hong et al., 2016). Taken together, these studies suggest that activity blockade could cause a Rab3A-dependent inhibition of BDNF release, leading to an increase in postsynaptic receptors. However, we have no evidence that Rab3A is required for the regulation of BDNF release from neurons, so it is just as likely that after activity blockade, Rab3A acts in a positive way to promote release of a trophic molecule that stimulates the insertion of postsynaptic receptors. It is unlikely that Rab3A is required when activity is normal because receptor levels do not change in the same way with loss of Rab3A as they do with loss of activity. Rather, our data fit with a model in which Rab3A mainly becomes engaged in response to activity blockade (Figure 9).

### Is Rab3A acting in the postsynaptic cell to cause homeostatic increase in mEPSC amplitude?

To our knowledge, there is no evidence localizing Rab3A to postsynaptic sites. Other presynaptic molecules, such as SNARE proteins, synaptotagmins, and NSF, have been identified in the postsynaptic compartment (Lledo et al., 1998; Nishimune et al., 1998; Osten et al., 1998; Song et al., 1998; Araki et al., 2010; Kennedy et al., 2010; Suh et al., 2010; Jurado et al., 2013; Hussain and Davanger, 2015; Gu et al., 2016; Wu et al., 2017) but Rab3A localization was not examined in those studies. On the other hand, a recent report implicates Rab3A in the localization of plasma membrane proteins in rafts in HEK cells and Jurkat-T cells, indicating Rab3A can be found in locations other than the presynaptic nerve terminal (Diaz-Rohrer et al., 2023). In this study a reduction in all Rab3 isoforms led to an impairment of plasma membrane localization of epidermal growth factor receptor (EGFR), so it is possible that Rab3A is acting similarly in postsynaptic dendrites to promote transport of GluA2 receptors to the surface after activity blockade (Figure 9). Another possibility, not depicted in Figure 9, is that Rab3A acts postsynaptically to regulate the release of a trophic factor that in turn regulates surface expression of GluA2 receptors. This idea is consistent with evidence that BDNF is synthesized and released locally from dendrites (Jakawich et al., 2010b; Thapliyal et al., 2022).

### Alternate contributors to homeostatic increase in mEPSC amplitude

We do not think Rab3A impacts homeostatic synaptic plasticity solely through pre- or postsynaptic regulation of GluA2 receptor trafficking to the plasma membrane. First, prolonged activity blockade with TTX at the NMJ led to an increase in mEPC amplitude that was not accompanied by any increase in AChR levels, yet it was dependent on Rab3A (Wang et al., 2011). Second, mEPSC amplitudes increased in three cortical cultures treated with TTX, but GluA2 receptor levels in one of the cultures actually went down. An increase in conductance could explain the increase in mEPSC amplitudes in the absence of receptor level increases, but all evidence to date suggests that the only way to increase conductance is to either switch from GluA2-containing heteromeric receptors to GluA1 homomers, which have much greater conductance (Oh and Derkach, 2005; Soares et al., 2013; Hou et al., 2015; Xu and Pozzo-Miller, 2017; Benke and Traynelis, 2019; Silva et al., 2019; Dubes et al., 2022), or, to post-translationally modify the receptor. Such conductance-altering modifications have been demonstrated only for GluA1 (Derkach et al., 1999; Kristensen et al., 2011; Jenkins and Traynelis, 2012). Importantly, any change in conductance due to an increase in GluA1 homomers or modification of GluA1 should have been detected by NASPM being able to disrupt the homeostatic increase in mEPSC amplitude, and this was not what we observed.

The other contributor to mEPSC amplitude besides receptors is the size of the presynaptic quantum, or the amount of transmitter released during a single fusion event. As stated above, we found a downward trend in VGLUT1 levels after activity blockade. This downward trend was not apparent in cultures prepared from Rab3A^−/−^ mice, suggesting a possible Rab3A-dependent effect. Interestingly, Rab3A levels are reduced 50% in hippocampal extracts from mice lacking VGLUT1 (Fremeau et al., 2004). Coupled with our finding that loss of Rab3A was associated with a highly significant decrease in VGLUT1 levels in untreated cultures, these results suggest an interdependence between Rab3A and VGLUT1. More importantly, our results rule out VGLUT1 levels as the source for increased mEPSC amplitude.

The amount of transmitter released during vesicle fusion can be affected by the kinetics of the fusion pore opening (Chang et al., 2017). We previously used amperometric measurements in mouse adrenal chromaffin cells to show loss of Rab3A increased the occurrence of very small amplitude fusion pore feet (Wang et al., 2008). Paired with our finding that there is an increase in the occurrence of very slow-rising, abnormally shaped mEPCs at the neuromuscular junction of Rab3A^−/−^ mice, our data suggest that small synaptic vesicles may have a fusion pore step under certain circumstances (see also (Chiang et al., 2018)). If activity blockade causes an increase in mEPSC amplitude due to a more rapid opening of a fusion pore, or a larger fusion pore conductance, it is possible that Rab3A is required for this modulation.

Finally, a simple way to increase the amount of transmitter released by a vesicle is to enlarge vesicle size. Larger vesicle diameter has been observed following activity disruption in multiple paradigms, including ocular deprivation, cortical ablation, and immobilization (Kawana et al., 1971; Lloret and Saavedra, 1975; Cheresharov et al., 1978). Loss of Rab3 family members is associated with increased vesicle size (Riedel et al., 2002; Schonn et al., 2010). Finally, an increase in volume has been shown to increase miniature endplate junctional current amplitude at the Drosophila NMJ (Karunanithi et al., 2002). Rab3A could be required for an activity-dependent increase in vesicle size, but the increase in diameter needed for a 25% increase in transmitter, and therefore mEPSC amplitude, would be difficult to detect.

To our knowledge, this is the first evidence of a presynaptic protein being implicated in the homeostatic regulation of mEPSC amplitude and AMPARs in neuronal cultures. Homeostatic synaptic plasticity is becoming increasingly implicated in essential normal functions such as sleep (Tononi and Cirelli, 2014; Diering et al., 2017; Torrado Pacheco et al., 2021), and pathological conditions such as epilepsy, neuropsychiatric disorders, Huntington’s, and alcohol use disorder (Trasande and Ramirez, 2007; Fernandes and Carvalho, 2016; Wang et al., 2017; Lovinger and Abrahao, 2018; Smith-Dijak et al., 2019; Lignani et al., 2020; Suzuki et al., 2021; Kavalali and Monteggia, 2023). Therefore our identification of a novel regulatory pathway involving Rab3A may have important therapeutic implications. In future work it will be important to determine whether Rab3A is acting presynaptically or postsynaptically to regulate GluA2 receptors, and what other contributor to mEPSC amplitude depends on Rab3A, possibly fusion pore characteristics or vesicle volume.

## Materials and Methods

### Animals.

To determine the role of Rab3A in homeostatic plasticity in mouse cortical cultures, we employed two distinct genetic mouse strains with altered Rab3A function. Rab3A deletion mice were generated as follows: Rab3A^+/−^ heterozygous mice were bred and genotyped as previously described (Kapfhamer et al., 2002; Wang et al., 2008), and maintained as a colony using multiple heterozygous breeding pairs. Homozygous Rab3A^+/+^ or Rab3A^−/−^ mouse pups were obtained by breeding Rab3A^+/+^ pairs and Rab3A^−/−^ pairs, respectively, so that the pups were the first generation progeny of a homozygous mating. This protocol minimizes the tendency of multiple generations of Rab3A^+/+^ and Rab3A^−/−^ homozygous breedings to produce progeny that are more and more genetically distinct. Rab3A^*Ebd/Ebd*^ mice were identified in an EU-mutagenesis screen of C57BL/6J mice, and after a cross to C3H/HeJ, were backcrossed for 3 generations to C57BL/6J (Kapfhamer et al., 2002). Rab3A^+/*Ebd*^ heterozygous mice were bred at Wright State University and genotyped in a two-step procedure: 1. a PCR reaction (forward primer: TGA CTC CTT CAC TCC AGC CT; reverse primer: TGC ACT GCA TTA AAT GAC TCC T) followed by 2. a restriction digest with enzyme Bsp1286I (New England Biolabs) that distinguishes the Earlybird mutant by its different base-pair products. Rab3A^+/−^ mice were backcrossed with Rab3A^+/+^ mice from the Earlybird heterozygous colony for 11 generations in an attempt to establish a single wild type strain, but differences in mEPSC amplitude and adrenal chromaffin cell calcium currents persisted, likely due to genes that are close to the Rab3A site, resulting in two wild type strains: 1. Rab3A^+/+^ from the Rab3A^+/−^ colony, and 2. Rab3A^+/+^ from the Rab3A^+/*Ebd*^ colony.

### Primary Culture of Mouse Cortical Neurons.

Primary dissociated cultures of mixed neuronal and glia populations were prepared as previously described (Hanes et al., 2020). Briefly, postnatal day 0–2 (P0-P2) Rab3A^+/+^, Rab3A^−/−^ or Rab3A^*Ebd/Ebd*^ neonates were euthanized by rapid decapitation, as approved by the Wright State University Institutional Animal Care and Use Committee, and brains were quickly removed. Each culture was prepared from the cortices harvested from two animals; neonates were not sexed. Cortices were collected in chilled Neurobasal-A media (Gibco) with osmolarity adjusted to 270 mOsm and supplemented with 40 U/ml DNAse I (ThermoFisher Scientific). The tissues were digested with papain (Worthington Biochemical) at 20 U/ml at 37°C for 20 minutes followed by trituration with a sterile, fire-polished Pasteur pipette, then filtered through a 100 μm cell strainer, and centrifuged at 1100 rpm for 2 minutes. After discarding the supernatant, the pellet was resuspended in room temperature Neurobasal-A media (270 mOsm), supplemented with 5% fetal bovine serum for glia growth, and 2% B-27 supplement to promote neuronal growth (Gibco), L-glutamine, and gentamicin (ThermoFisher Scientific). Neurons were counted and plated at 0.15 * 10^6^ cells/coverslip onto 12 mm coverslips pre-coated with poly-L-lysine (BioCoat, Corning). This plating density produces a “high density” culture characterized by a complex mesh of neuronal processes criss-crossing the field of view, completely filling the space between cell bodies (see Figure 4). We found in preliminary experiments that an increase in mEPSC amplitude in mouse cortical cultures was inconsistent when cell density was low, likely due to lower levels of baseline activity, although it is a limitation of this study that we did not directly measure activity levels in cultures prepared from Rab3A^+/+^, Rab3A^−/−^ or Rab3A^Ebd/Ebd^ mice, and therefore do not know if differences in results are due to differences in activity levels. While unlikely for Rab3A^+/+^ and Rab3A^−/−^ studies, given the mEPSC amplitude in untreated cultures that was relatively small, it remains possible that in Rab3A^Ebd/Ebd^ studies, the larger mEPSC amplitude in untreated cultures was due to a loss of activity in these cultures. The culture media for the first day (0 DIV) was the same as the above Neurobasal-A media supplemented with FBS, B-27, L-glutamine, and gentamicin, and was switched after 24 hours (1 DIV) to media consisting of Neurobasal-A (270 mOsm), 2% B-27, and L-glutamine without FBS to avoid its toxic effects on neuronal viability and health (Stellwagen and Malenka, 2006). Half of the media was changed twice weekly and experiments were performed at 13–14 DIV. Two days prior to experiments, tetrodotoxin (TTX) (500 nM; Tocris), a potent Na^+^ channel blocker, was added to some cultures to chronically silence all network activity and induce homeostatic synaptic plasticity mechanisms, while untreated sister cultures served as controls. Cultures prepared from mutant mice were compared with cultures from wild-type mice from their respective colonies. Note that the cultures comprising the Rab3A^+/+^ data here are a subset of the data previously published in Hanes et al., 2020. This smaller data set was restricted to the time period over which cultures were also prepared from Rab3A^−/−^ mice.

### Preparation of Glial Feeder Layers.

Glial feeder layers were prepared from the cortices of P0-P2 Rab3A^+/+^ or Rab3A^−/−^ mouse pups as described previously (Stellwagen and Malenka, 2006). Briefly, cortices were dissected and cells were dissociated as described above. Cell suspensions of mixed neuronal and glial populations were plated onto glass coverslips pre-coated with poly-L-lysine in Dulbecco’s Modified Eagle Media (ThermoFisher Scientific) supplemented with 5% FBS (to promote glial proliferation and to kill neurons), L-glutamine, and gentamicin, and maintained in an incubator at 37°C, 5% CO_2_; cultures were maintained in this manner for up to 1 month to generate purely glial cultures (all neurons typically died off by 7 DIV). Culture media was replaced after 24 hours, and subsequent media changes were made twice weekly, replacing half of the culture media with fresh media. Feeder layers were not used for neuronal seeding until all native neurons were gone and glia approached 100% confluency (visually inspected).

### Plating of Neurons on Glial Feeder Layers.

Cortical neurons were obtained as described above. The cell pellet obtained was resuspended in Neurobasal-A (osmolarity adjusted to 270 mOsm) containing B27 (2%, to promote neuronal growth), L-glutamine, and 5-fluorodeoxyuridine (FdU, a mitotic inhibitor; Sigma). Addition of FdU was used to prevent glial proliferation and contamination of the feeder layer with new glia, promoting only neuronal growth on the feeder layers (FdU-containing media was used for the maintenance of these cultures and all subsequent media changes). Glial culture media was removed from the feeder layer cultures, and the neuronal cell suspension was plated onto the glial feeder cultures. The culture strategy used to distinguish the relative roles of neuronal and glial Rab3A is outlined in Figure 8, Left. At 1 DIV, all of the culture media was removed and replaced with fresh Neurobasal-A media containing FdU described above, and half of the media was replaced twice per week for all subsequent media changes. Cultures were maintained in a 37°C, 5% CO_2_ incubator for 13–14 DIV.

### Whole-Cell Voltage Clamp to Record mEPSCs.

At 13–14 DIV, mEPSCs from TTX-treated and untreated sister cultures of Rab3A^+/+^ or Rab3A^−/−^ neurons from the Rab3A^+/−^ colony, or Rab3A^+/+^ or Rab3A^*Ebd/Ebd*^ neurons from the Rab3A^+/*Ebd*^ colony, were recorded via whole-cell voltage clamp to assess the role of Rab3A in homeostatic synaptic plasticity. Recordings were taken from pyramidal neurons, which were identified visually by a prominent apical dendrite; images were taken of all cells recorded from. Cells were continuously perfused with a solution consisting of (in mM): NaCl (115), KCl (5), CaCl_2_ (2.5), MgCl_2_ (1.3), dextrose (23), sucrose (26), HEPES (4.2), pH = 7.2 (Stellwagen and Malenka, 2006). On the day of recording, the osmolarity of the media from the cultures was measured (normally 285 – 295 mOsm) and the perfusate osmolarity was adjusted to match the culture osmolarity, to protect against osmotic shock to the neurons. To isolate glutamatergic mEPSCs, TTX (500 nM) and picrotoxin (50 μM) were included in the perfusion solution to block action potentials and GABAergic currents, respectively. The NMDA receptor antagonist, APV, was not included in the perfusion solution because in pilot studies, all mEPSCs were blocked by 10 μM CN0X and 50 μm picrotoxin, demonstrating no APV-sensitive mEPSCs were present (data not shown). Previous studies have described NMDA mEPSCs in neuronal cultures, but recordings were performed in 0 Mg^2+^ (Watt et al., 2000; Sutton et al., 2006). In our recordings, the presence of extracellular Mg^2+^ (1.3 mM) and TTX would mean that the majority of NMDA receptors are blocked by extracellular Mg^2+^ at resting Vm. Patch electrodes (3.5 – 5 M0) were filled with an internal solution containing (in mM): K-gluconate (130), NaCl (10), EGTA (1), CaCl_2_ (0.132), MgCl_2_ (2), HEPES (10), pH = 7.2. Osmolarity was adjusted to 10 mOsm less than the perfusion solution osmolarity, which we have found improves success rate of achieving low access resistance recordings. Neurons were clamped at a voltage of −60 mV using an Axopatch 200B patch-clamp (Axon Instruments), recorded from for 2 – 5 minutes, and data were collected with Clampex 10.0/10.2 (Axon Instruments).

### NASPM application.

The antagonist of Ca^2+^-permeable AMPA receptors, N-naphthyl acetylspermine (NASPM, 20 μM; Tocris), was applied during recordings in a subset of experiments. NASPM is a synthetic analog of Joro Spider Toxin (JSTX) (Koike et al., 1997), and chemicals in this family block Ca^2+^ permeable glutamate receptors (Blaschke et al., 1993; Herlitze et al., 1993; lino et al., 1996). The presence of the edited GluA2 subunit disrupts both the Ca^2+^ permeability and the sensitivity to JSTX and related substances (Blaschke et al., 1993; Bochet et al., 1994; Jonas and Burnashev, 1995). Therefore, 20 μM NASPM is expected to completely block AMPA receptors containing GluA1, GluA3 or GluA4 subunits (Hollmann et al., 1991; Burnashev et al., 1992), or the Kainate receptors containing GluA5 or GluA6 (Koike et al., 1997; Sun et al., 2009), but be ineffective against any heteromer or homomer containing GluA2 subunits. Because the effect of the spider toxins and their analogues are use-dependent (Herlitze et al., 1993; lino et al., 1996; Koike et al., 1997), NASPM was applied with a mildly depolarizing high K^+^ solution (25 mM KCl, 95 mM NaCl) which we expected to trigger release of glutamate and opening of glutamate-activated receptors, allowing entry of the pore blocker. Baseline recordings were performed for 2 minutes in our standard perfusate, then were suspended while NASPM + High K^+^ solution was applied for 45 seconds, followed by a NASPM only solution for 5 minutes, after which recording was recommenced for 5 minutes (because we found in pilot experiments that frequency was reduced following NASPM application).

### Analysis of mEPSCs.

Miniature excitatory postsynaptic currents (mEPSCs) were manually selected using Mini Analysis software (Synaptosoft), a standard program used for mEPSC event detection and analysis. Records were filtered at 2 kHz using a low-pass Butterworth filter prior to selection. The program threshold was set at 3 pA but the smallest mEPSC selected was 4.01 pA. MiniAnalysis selects many false positives with the automated feature when a small threshold amplitude value is employed, due to random fluctuations in noise, so manual re-evaluation of the automated process is necessary to eliminate false positives. If the threshold value is set high, there are few false positives but small amplitude events that visually are clearly mEPSCs are missed, and manual re-evaluation is necessary to add back false negatives or the population ends up biased towards large mEPSC amplitudes. As soon as there is a manual step, bias is introduced. Interestingly, a manual reevaluation step was applied in a recent study that describes their process as ‘unbiased’ (Wu et al., 2020). In sum, we do not believe it is currently possible to perform a completely unbiased detection process. A fully manual detection process means that the same criterion (“does this *look* like an mEPSC?”) is applied to all events, not just the false positives, or the false negatives, which prevents the bias from being primarily at one end or the other of the range of mEPSC amplitudes. It is important to note that when performing the MiniAnalysis process, the researcher did not know whether a record was from an untreated cell or a TTX-treated cell.

### Immunofluorescence, microscopy, and data analysis.

Primary cultures of mouse cortical neurons were grown for 13–14 DIV. Antibodies to GluA2 (mouse ab against N-terminal, EMD Millipore) were added directly to live cultures at 1:40 dilution, and incubated at 37 °C in a CO_2_ incubator for 45 minutes. Cultures were rinsed 3 times with PBS/5% donkey serum before being fixed with 4% paraformaldehyde. After 3 rinses in PBS/5% donkey serum, cultures were incubated in CY3-labeled donkey-anti-mouse secondary antibodies for 1 hour at room temperature, rinsed in PBS/5% donkey serum, permeabilized with 0.2% saponin, and incubated in chick anti-MAP2 (1:2500, AbCAM) and rabbit anti-VGLUT1 (1:4000, Synaptic Systems) for 1 hour at room temperature in PBS/5% donkey serum. After rinsing with PBS/5% donkey serum, coverslips were incubated with 488-anti chick and CY5-anti rabbit secondary antibodies for 1 hour at room temperature, rinsed, blotted to remove excess liquid, and inverted on a drop of Vectashield (Vector Labs). Coverslips were sealed with nail polish and stored at 4 °C for < 1 week before imaging. All secondary antibodies were from Jackson Immunoresearch and were used at 1:225 dilution.

Coverslips were viewed on a Fluoview FV1000 laser scanning confocal microscope with a 60x oil immersion, 1.35 NA objective. Once a pyramidal neuron was identified and a single confocal section of the cell acquired (see Top, Figure 4), Fluoview 2.1 software was used to zoom in on the primary dendrite (5X) and a series of confocal sections were taken every 0.5 μm. Images were analyzed offline with ImagePro 6 (Cybernetics). The composite image was used to locate synaptic sites containing both VGLUT1 and GluA2 immunoreactivity in close apposition to each other and to the primary dendrite or a secondary branch identified with MAP2 immunoreactivity. An area of interest (AOI) was manually drawn around the GluA2 cluster in the confocal section in which it was the brightest. The AOIs for a dendrite were saved in a single file; the AOI number and the confocal section it was associated with were noted for later retrieval. For quantification, AOIs were loaded, an individual AOI was called up on the appropriate section, and the count/measurement tool used to apply a threshold. Multiple thresholds were used, 400–550, but we found that within a given experiment, the magnitude of the TTX effect was not altered by different thresholds, therefore, all data presented here use a threshold of 450. Pixels within the cluster that were above the threshold were automatically outlined, and size, average intensity, and integral of the outlined region saved to Origin2020 (OriginLab) file for calculating mean values. For analyzing VGLUT1 sites, previous AOIs were examined, and if not directly over the VGLUT1 site, moved to encompass it (this happened when GluA2 and VGLUT1 were side by side rather than overlapping). The threshold was 200 for all VGLUT1 data except Rab3A^+/+^ Culture #1, where threshold was 400. There was no threshold that worked for both that culture and the other cultures, with too much of the background regions being included for Culture #1 with a threshold of 200, and too little of the synaptic site area being included for the other Cultures with a threshold of 400.

### Statistics.

In our previous publication (Hanes et al., 2020), we described the characteristics of homeostatic plasticity of mEPSC amplitudes in mouse cortical cultures using multiple mEPSC amplitude quantiles from each cell to create data sets with thousands of samples. We compared the cumulative distribution functions for mEPSC amplitudes in untreated vs. TTX-treated cultures, as well as sorting the values from smallest to largest, and plotted TTX values vs. control values in the rank order plot first described in (Turrigiano et al., 1998) to demonstrate synaptic scaling. Finally, we plotted the ratio of TTX values/control values vs. control values to show that the ratio, which represents the effect of TTX, increased with increasing control values, which we termed “divergent scaling.” Here, we wanted to avoid the inflation of sample size caused by pooling multiple mEPSCs per cell in order to statistically compare the effects of TTX in cultures of wild type mice, Rab3A^−/−^ mice and Rab3A^*Ebd/Ebd*^ mutant mice. Therefore, we computed the mean mEPSC amplitude for each cell and compared means from untreated and TTX-treated cultures with the non-parametric Kruskal-Wallis test, with N = the number of cells. The overall means are presented ± SEM in the text, and p values are displayed above each plot. The same process was carried out for the characteristics of synaptic GluA2 receptor clusters and VGLUT1 sites in imaging experiments, with N = the number of dendrites (one dendrite was sampled for each cell). Although we quantified three characteristics of the GluA2 clusters and VGLUT1 sites, size, average intensity, and integral, we have not corrected p values for multiple comparisons. Means are presented as box plots + data, with the box at 25 and 75 percentiles, whiskers at 10 and 90 percentiles, a dot indicating the mean, and a line indicating the median. In Figure 3, mEPSC amplitudes were measured within the same cell before and after NASPM application, so a paired T test was used to compare the pre- and post-NASPM mEPSC amplitudes. Statistics were computed and plots were created using Origin2020 (OriginLab).

## Figures and Tables

**Table 1.** Comparison of mEPSC amplitude and GluA2 receptor cluster characteristics in the same mouse cortical cultures prepared from Rab3A^+/+^ mice, and another set of cultures prepared from Rab3A^−/−^ mice. Data pooled from 3 cultures, and data from each of the 3 individual cultures.

	Rab3A^+/+^ Pooled Data from 3 cultures				Rab3A^−/−^ Pooled Data from 3 cultures			
Quantity	CON Mean, SE (N)	TTX Mean, SE (N)	% change	p value	CON Mean, SE (N)	TTX Mean, SE (N)	% change	p value
mEPSC amplitude	13.7 ± 0.9 pA (23)	16.4 ± 0.9 pA (24)	+19.7	*p = 0.02	14.9 ± 0.8 pA (21)	13.5 ± 0.9 pA (21)	−9.4	p = 0.18
GluA2 cluster size	0.90 ± 0.06 μm^2^ (29)	1.08 ± 0.10 μm^2^ (28)	+20.0	p = 0.27	0.93 ± 0.05 μm^2^ (30)	0.91 ± 0.05 μm^2^ (29)	−2.2	p = 0.75
GluA2 cluster intensity	684 ± 15 a.u. (29)	699 ± 12 a.u. (28)	+2.2	p = 0.15	766 ± 12 a.u. (30)	776 ± 15 a.u. (29)	+1.3	p = 0.46
GluA2 cluster integral	317898 ± 26726 a.u. (29)	389487 ± 41465 a.u. (28)	+22.5	p = 0.26	369436 ± 23439 a.u. (30)	364237 ± 25833 a.u. (29)	−0.8	p = 0.86
Quantity	Rab3A^+/+^ Culture #1				Rab3A^−/−^ Culture #1 (not same culture as Rab3A^+/+^)			
mEPSC amplitude	14.2 ± 2.4 pA (6)	15.9 ± 1.9 pA (7)	+12.0	p = 0.25	15.4 ± 1.2 pA (7)	14.4 ± 0.8 pA (6)	−6.5	p = 0.20
GluA2 cluster size	0.73 ± 0.09 μm^2^ (10)	0.89 ± 0.09 μm^2^ (10)	+21.9	p = 0.45	0.87 ± 0.07 μm^2^ (10)	0.80 ± 0.12 μm^2^ (10)	−8.0	p = 0.41
GluA2 cluster intensity	682 ± 15 a.u. (10)	704 ± 11 a.u. (10)	+3.2	p = 0.13	735 ± 10 a.u. (10)	731 ± 25 a.u.	−0.5	p = 0.71
GluA2 cluster integral	256364 ± 35242 a.u. (10)	316647 ± 35077 a.u. (10)	+23.5	p = 0.29	328477 ± 28250 a.u. (10)	302594 ± 53052 a.u. (10)	−7.9	p = 0.41
Quantity	Rab3A^+/+^ Culture #2				Rab3A^−/−^ Culture #2 (not the same culture as Rab3A^+/+^)			
mEPSC amplitude	13.8 ± 2.2 pA (7)	17.7 ± 1.8 pA (8)	+28.2	p = 0.16	15.2 ± 1.8 pA (8)	13.5 ± 1.8 pA (9)	−11.2	p = 0.50
GluA2 cluster size	0.91 ± 0.12 μm^2^ (9)	1.42 ± 0.24 μm^2^ (9)	+56.0	*p = 0.05	1.05 ± 0.10 μm^2^ (10)	0.91 ± 0.04 μm^2^ (9)	−13.3	p = 0.37
GluA2 cluster intensity	621 ± 14 a.u. (9)	679 ± 26 a.u. (9)	+9.3	*p = 0.04	800 ± 26 a.u. (10)	782 ± 23 a.u. (9)	−2.2	p = 0.51
GluA2 cluster integral	296300 ± 44108 a.u. (9)	512336 ± 96892 a.u. (9)	+72.9	*p = 0.04	425552 ± 49486 a.u. (10)	354289 ± 23099 a.u. (9)	−16.7	p = 0.33
Quantity	Rab3A^+/+^ Culture #3				Rab3A^−/−^ Culture #3 (not the same culture as Rab3A^+/+^)			
mEPSC amplitude	13.4 ± 0.8 pA (10)	15.7 ± 1.0 pA (9)	+17.2	p = 0.10	13.9 ± 1.2 pA (6)	12.9 ± 1.5 pA (6)	−7.2	p = 0.26
GluA2 cluster size	1.05 ± 0.11 μm^2^ (10)	0.95 ± 0.15 μm^2^ (9)	−9.5	p = 0.46	0.89 ± 0.08 μm^2^ (10)	1.03 ± 0.08 μm^2^ (10)	+15.7	p = 0.29
GluA2 cluster intensity	744 ± 28 a.u. (10)	714 ± 25 A.U. (9)	−4.0	p = 0.29	765 ± 22 a.u. (10)	814 ± 23 a.u. (10)	+6.4	p = 0.17
GluA2 cluster integral	398870 ± 49500 a.u. (10)	347571 ± 65454 a.u. (9)	−12.9	p = 0.46	354279 ± 38755 a.u. (10)	434833 ± 42350 a.u. (10)	+22.7	p = 0.29

**Table 2.** Comparison of mEPSC amplitude and VGLUT1 positive presynaptic site characteristics in the same mouse cortical cultures prepared from Rab3A^+/+^ and Rab3A^−/−^ mice. Data pooled from 3 cultures, and data from individual cultures. mEPSC data are identical to that in Table 1, reproduced here for comparison purposes.

	Rab3A^+/+^ Pooled Data from 3 cultures				Rab3A^−/−^ Pooled Data from 3 cultures			
Quantity	CON Mean, SE (N)	TTX Mean, SE (N)	% change	p value	CON Mean, SE (N)	TTX Mean, SE (N)	% change	p value
mEPSC amplitude	13.7 ± 0.9 pA (23)	16.4 ± 0.9 pA (24)	+19.7	*p = 0.02	14.9 ± 0.8 pA (21)	13.5 ± 0.9 pA (21)	−9.4	p = 0.18
VGLUT1 site size	1.17 ± 0.08 μm^2^ (29)	1.07 ± 0.06 μm^2^ (24)	−7.7	p = 0.97	0.89 ± 0.03 μm^2^ (30)	0.95 ± 0.04 μm^2^ (29)	+6.7	p = 0.55
VGLUT1 site intensity	699 ± 39 a.u. (29)	639 ± 31 a.u. (28)	−8.6	p = 0.28	554 ± 13 a.u. (30)	570 ± 19 a.u. (29)	+2.9	p = 0.18
VGLUT1 site integral	430787 ± 36818 a.u. (29)	351653 ± 21689 a.u. (28)	−18.4	p = 0.13	257159 ± 18265 a.u. (30)	289857 ± 18521 a.u. (29)	+12.7	p = 0.21
Quantity	Rab3A^+/+^ Culture #1				Rab3A^−/−^ Culture #1 (not same culture as Rab3A^+/+^)			
mEPSC amplitude	14.2 ± 2.4 pA (6)	15.9 ± 1.9 pA (7)	+12.0	p = 0.25	15.4 ± 1.2 pA (7)	14.4 ± 0.8 pA (6)	−6.5	p = 0.20
VGLUT1 site size	0.86 ± 0.06 μm^2^ (10)	0.79 ± 0.04 μm^2^ (10)	−8.1	p = 0.11	0.85 ± 0.04 μm^2^ (10)	1.05 ± 0.07 μm^2^ (10)	+23.5	p = 0.15
VGLUT1 site intensity	919 ± 35 a.u. (10)	832 ± 18 a.u. (10)	−9.5	p = 0.10	526 ± 17 a.u. (10)	629 ± 33 a.u. (10)	+19.6	p = 0.10
VGLUT1 site integral	445647 ± 39114 a.u. (10)	355055 ± 22354 a.u. (10)	−20.3	p = 0.07	233589 ± 16145 a.u. (10)	329851 ± 32856 a.u. (10)	+41.2	p = 0.10
Quantity	Rab3A^+/+^ Culture #2				Rab3A^−/−^ Culture #2 (not same culture as Rab3A^+/+^)			
mEPSC amplitude	13.8 ± 2.2 pA (7)	17.7 ± 1.8 pA (8)	+28.2	p = 0.16	15.2 ± 1.8 pA (8)	13.5 ± 1.8 pA (9)	−11.2	p = 0.50
VGLUT1 site size	0.95 ± 0.09 μm^2^ (9)	1.01 ± 0.05 μm^2^ (9)	+6.3	p = 0.35	0.81 ± 0.05 μm^2^ (10)	0.82 ± 0.03 μm^2^ (9)	+1.2	p = 0.68
VGLUT1 site intensity	454 ± 19 a.u. (9)	459 ± 34 a.u. (9)	+1.1	p = 0.57	505 ± 14 a.u. (10)	538 ± 19 a.u. (9)	+6.1	p = 0.29
VGLUT1 site integral	239671 ± 29790 a.u. (9)	253222 ± 35117 a.u. (9)	+5.6	p = 0.96	213066 ± 19080 a.u. (10)	236927 ± 12964 a.u. (9)	+11.2	p = 0.41
Quantity	Rab3A^+/+^ Culture #3				Rab3A^−/−^ Culture #3 (not same culture as Rab3A^+/+^)			
mEPSC amplitude	13.4 ± 0.8 pA (10)	15.7 ± 1.0 pA (9)	+17.2	p = 0.10	13.9 ± 1.2 pA (6)	12.9 ± 1.5 pA (6)	−7.2	p = 0.26
VGLUT1 site size	1.64 ± 0.13 μm^2^ (10)	1.41 ± 0.10 μm^2^ (10)	−13.4	p = 0.17	1.01 ± 0.05 μm^2^ (10)	0.97 ± 0.07 μm^2^ (10)	−4.0	p = 0.76
VGLUT1 site intensity	700 ± 33 a.u. (10)	608 ± 12 a.u. (10)	−13.1	*p = 0.02	568 ± 31 a.u. (10)	538 ± 35 a.u. (10)	−5.3	p = 0.55
VGLUT1 site integral	587932 ± 59742 a.u. (10)	436840 ± 31327 a.u. (10)	−25.7	*p = 0.05	324823 ± 42586 a.u. (10)	297499 ± 37637 a.u. (10)	−8.4	p = 0.82
