# Supplementary material for: Homeostatic Synaptic Plasticity of Miniature Excitatory Postsynaptic Currents in Mouse Cortical Cultures Requires Neuronal Rab3A
Source: bioRxiv. 2024 Dec 4:2023.06.14.544980. Preprint. [Version 3] doi: 10.1101/2023.06.14.544980 (PMC11275788; doi:10.1101/2023.06.14.544980)
Supplement: Supplementary file 1 [file NIHPP2023.06.14.544980v3-supplement-1.pdf]

bioRxiv preprint doi: https://doi.org/10.1101/2023.06.14.544980; this version posted December 4, 2024. The copyright holder for this preprint (which was not certified by peer review) is the author/funder, who has granted bioRxiv a license to display the preprint in perpetuity. It is made available under aCC-BY 4.0 International license.

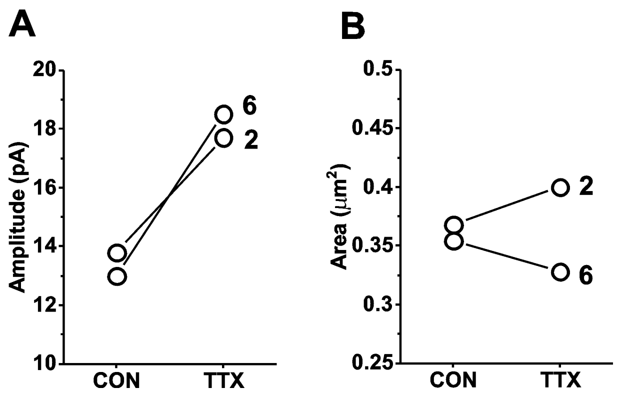

Supplemental Figure 1. Comparison of mEPSC amplitudes and GluA1 receptor cluster areas in matched mouse cortical cultures prepared from Rab3A$^{+/+}$ mice and treated with TTX for 48 hr.

(A) Culture averages of mEPSC amplitudes for untreated (CON) and TTX-treated coverslips (TTX) in each of 2 Rab3A$^{+/+}$ mouse cortical co-cultures. Culture #2, CON, N = 7, 13.8 $\pm$ 2.4 pA; TTX, N = 8, 17.7 $\pm$ 1.8 pA (+28.3%); Culture #6, CON, N = 6, 13.0 $\pm$ 1.5 pA; TTX, N = 6, 18.5 $\pm$ 2.0 pA (+42.3%). (B) Culture averages of GluA1 receptor cluster size in the same 2 cultures as shown in (A). Culture #2, CON, N = 10, 0.37 $\pm$ 0.04 μm^2; TTX, N = 10, 0.40 $\pm$ 0.06 μm^2 (+8.1%) Culture #6, CON, N = 10, 0.35 $\pm$ 0.05 μm^2; TTX, N = 10, 0.33 $\pm$ 0.04 μm^2 (-5.7%) “Culture #2” is the same Culture #2 depicted in Figure 5, different coverslips were processed for GluA1 immunofluorescence labeling. GluA1 receptor cluster intensity did not increase in Culture #2 (CON, 364 vs. TTX, 357) or Culture #6 (CON, 357 vs. TTX, 363). Live cultures were exposed to GluA1 antibody against the extracellular domain (ABN241, purchased from EMD Millipore, now available from Millipore Sigma) before being fixed and processed with secondary antibodies.
